# Supplementary material for: DNA methylation-based predictors of metabolic traits in Scottish and Singaporean cohorts
Source: Am J Hum Genet. 2024 Dec 19;112(1):106–15. doi: 10.1016/j.ajhg.2024.11.012 (PMC11739919; doi:10.1016/j.ajhg.2024.11.012)
Supplement: Document S1. Figures S1–S12 and supplemental methods [file mmc1.pdf]

The American Journal of Human Genetics, Volume *112*

Supplemental information

DNA methylation-based predictors of metabolic traits in Scottish and Singaporean cohorts

Hannah M. Smith, Hong Kiat Ng, Joanna E. Moodie, Danni A. Gadd, Daniel L. McCartney, Elena Bernabeu, Archie Campbell, Paul Redmond, Adele Taylor, Danielle Page, Janie Corley, Sarah E. Harris, Darwin Tay, Ian J. Deary, Kathryn L. Evans, Matthew R. Robinson, John C. Chambers, Marie Loh, Simon R. Cox, Riccardo E. Marioni, and Robert F. Hillary

A)

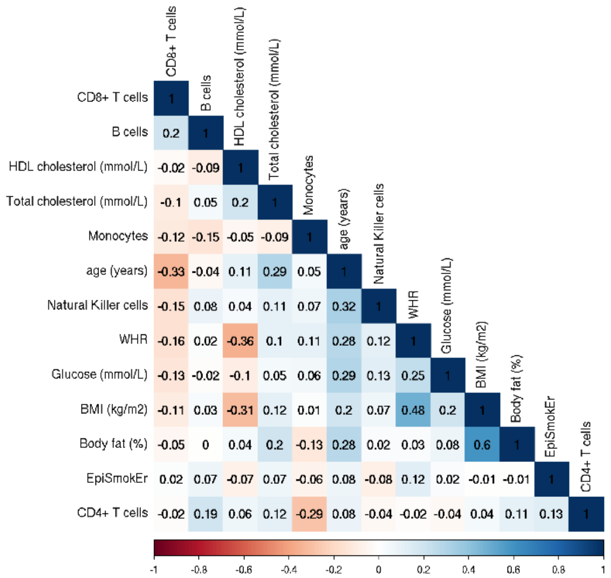

B)

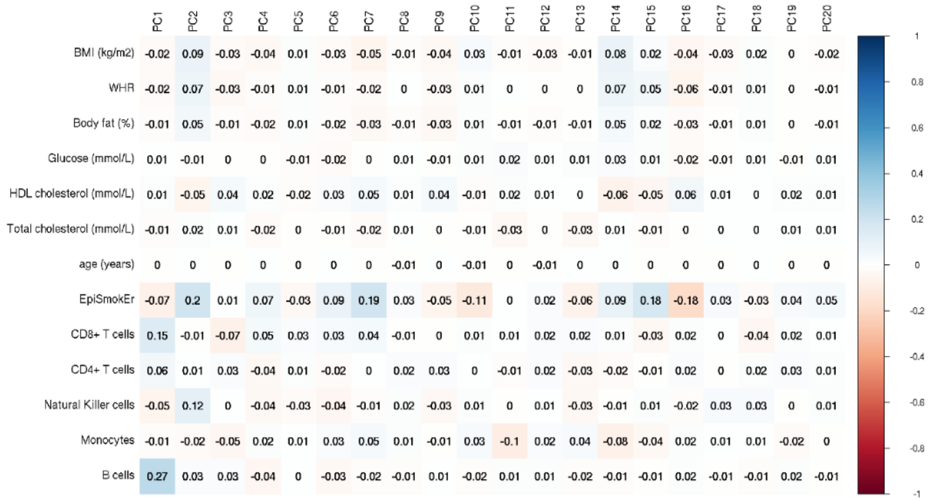

Figure S1: Metabolic trait, covariate and 20 DNAm PCs correlations in Generation Scotland. Figure S1A shows a heatmap of Pearson correlations between metabolic traits (BMI in kg/m^2; HDL cholesterol, total cholesterol and glucose in mmol/L; body fat in percentage; WHR) and covariates in Generation Scotland. Figure S1B shows a heatmap of Pearson correlations between metabolic traits/covariates and the first 20 DNAm PCs. BMI = body mass index; WHR = waist-hip ratio; HDL cholesterol = high-density lipoprotein cholesterol; EpiSmokEr = epigenetic smoking score; DNAm = DNA methylation; PCs = principal components.

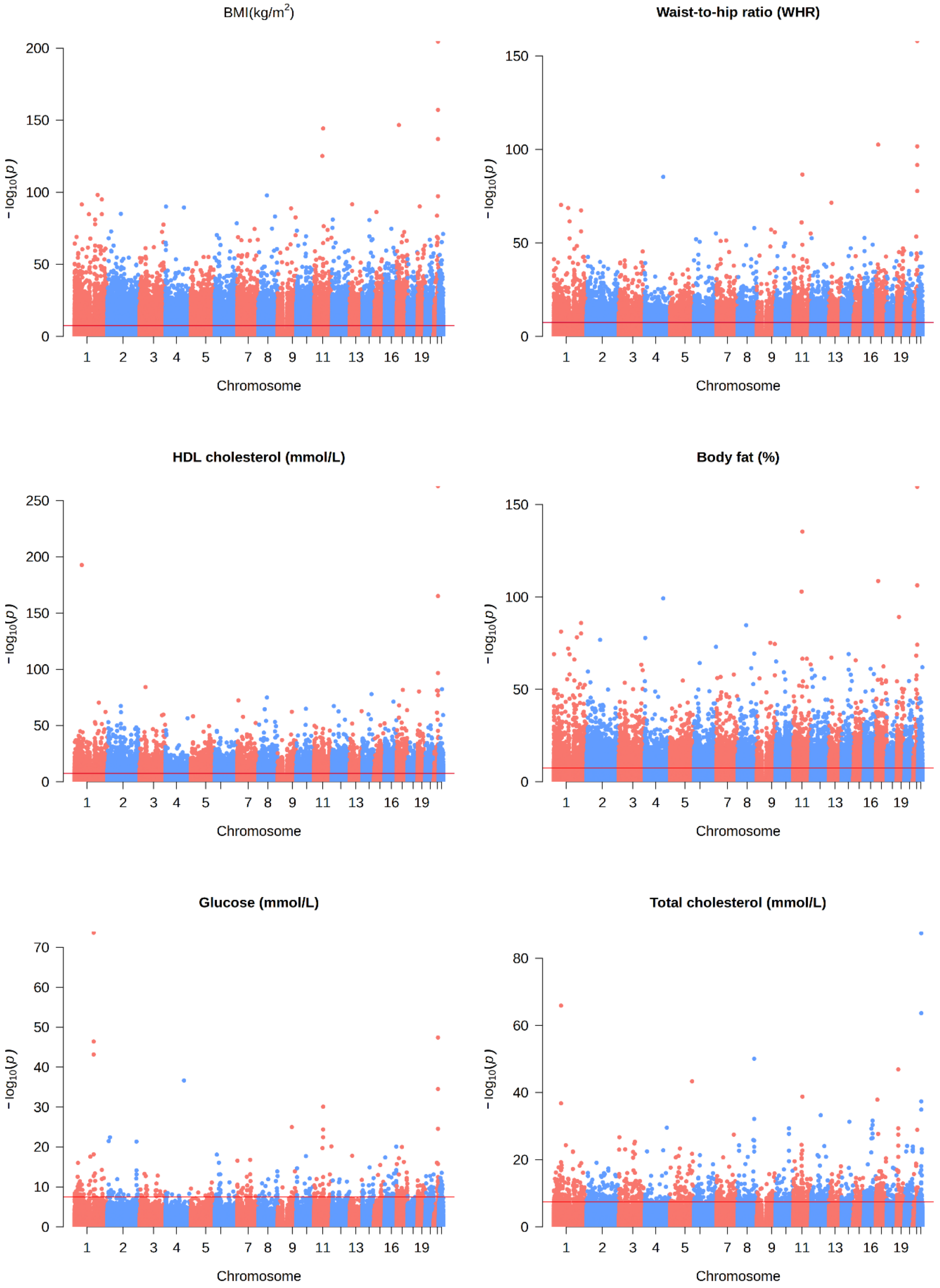

Figure S2: Manhattan plots of the results from the non-PC-adjusted marginal linear regression epigenome-wide association studies of six metabolic traits in Generation Scotland. The Manhattan plots for each of the six metabolic traits show each CpG as a data point. Outcomes in each EWAS are the residuals from metabolic traits regressed on age, age^2, sex and family structure. Original outcome units are indicated in the plot titles. The x-axis shows the chromosome position, and the y-axis shows the association significance (-$\log_{10}$(P)) for each CpG site. The horizontal red line indicates the significance threshold (P < 3.6 x 10^{-8}). BMI =

body mass index; WHR = waist-hip ratio; HDL cholesterol = high-density lipoprotein cholesterol; PC = principal component.

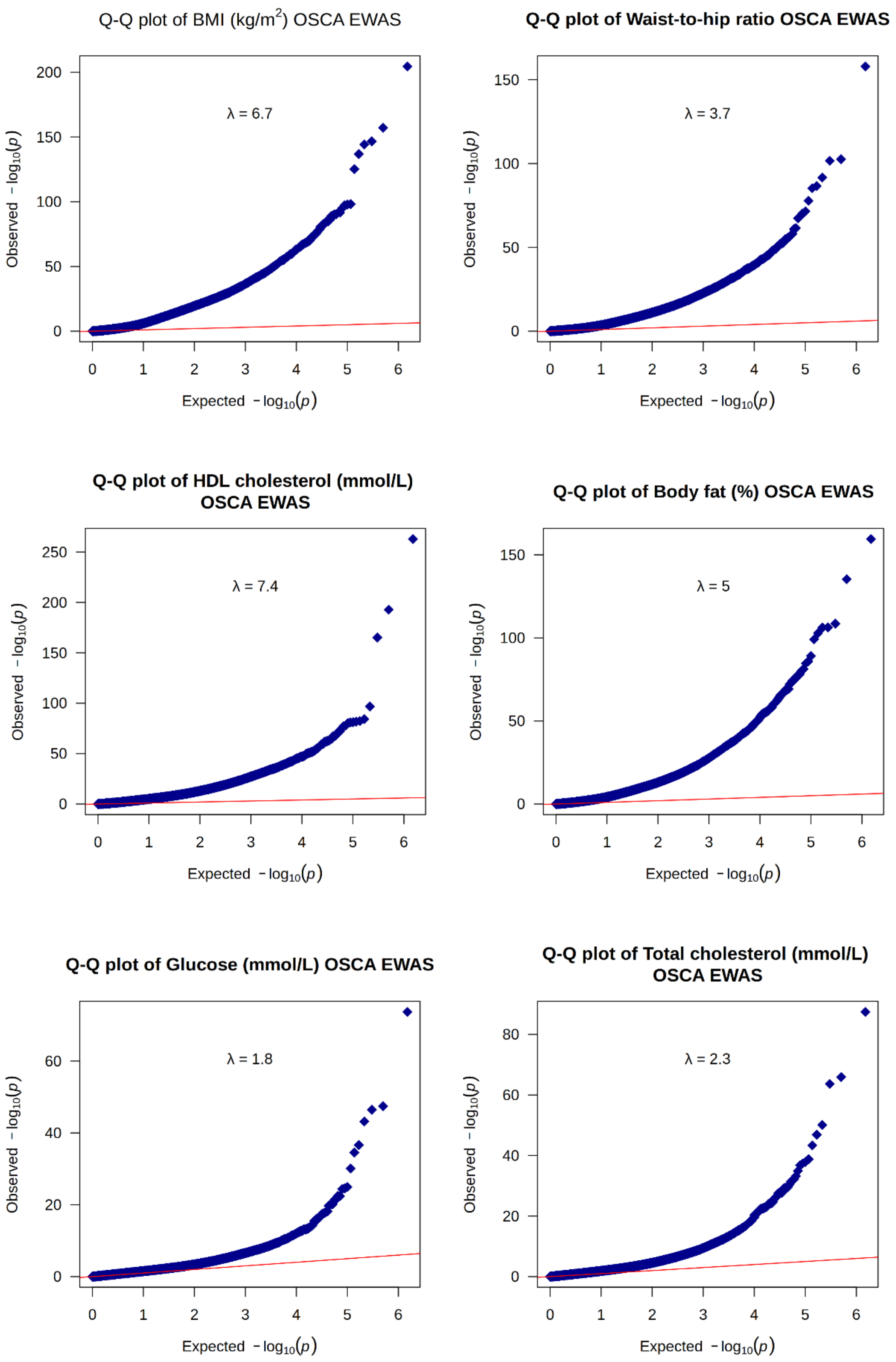

Figure S3: Quantile-Quantile plots of the results from the non-PC-adjusted marginal linear regression epigenome-wide association studies of six metabolic traits in Generation Scotland. The plots show expected $-\log_{10}(P)$ by the observed $-\log_{10}(P)$ for each metabolic trait. Outcomes in each EWAS are the residuals from metabolic traits regressed on age, age^2, sex and family structure. Original outcome units are indicated in the plot titles. The red line shows a trend line of where the observed and expected values are the same. The inflation factor, lambda (λ), is indicated on each plot. BMI = body mass index; WHR = waist-hip ratio; HDL cholesterol = high-density lipoprotein cholesterol; PC = principal component.

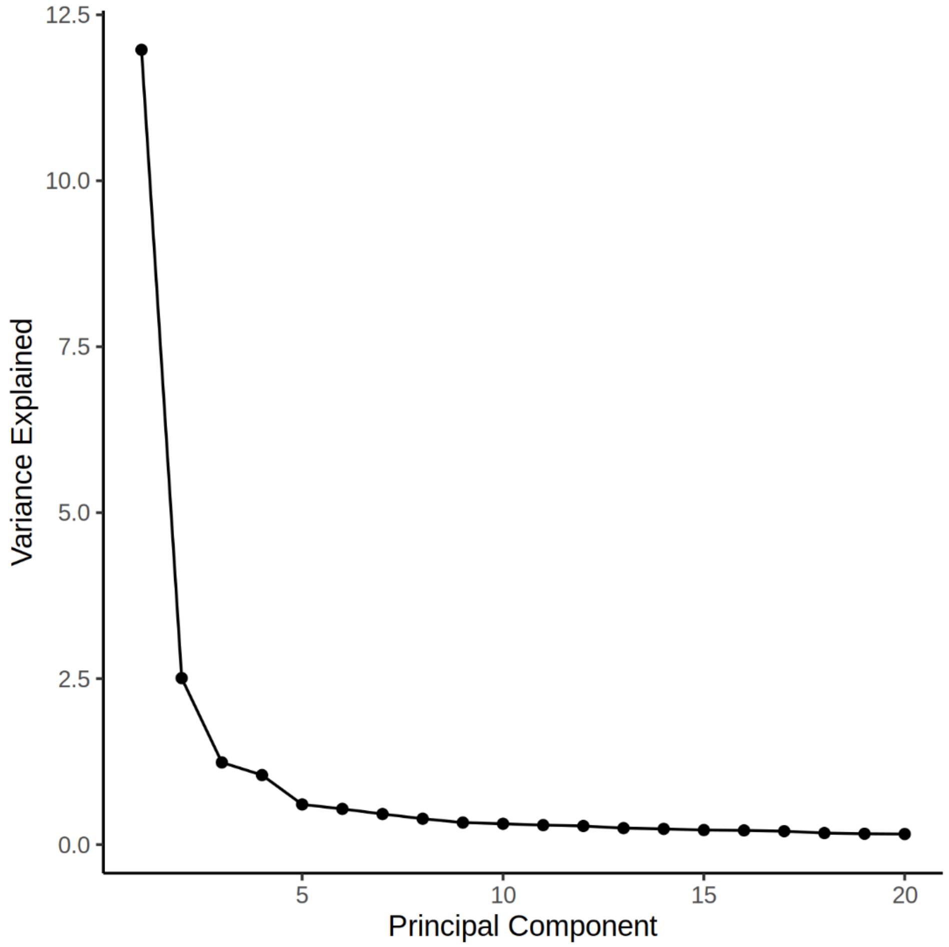

Figure S4: Variance explained in DNA methylation by the first 20 principal components. The plot shows the variance explained in the DNA methylation data by each of the first 20 principal components in Generation Scotland.

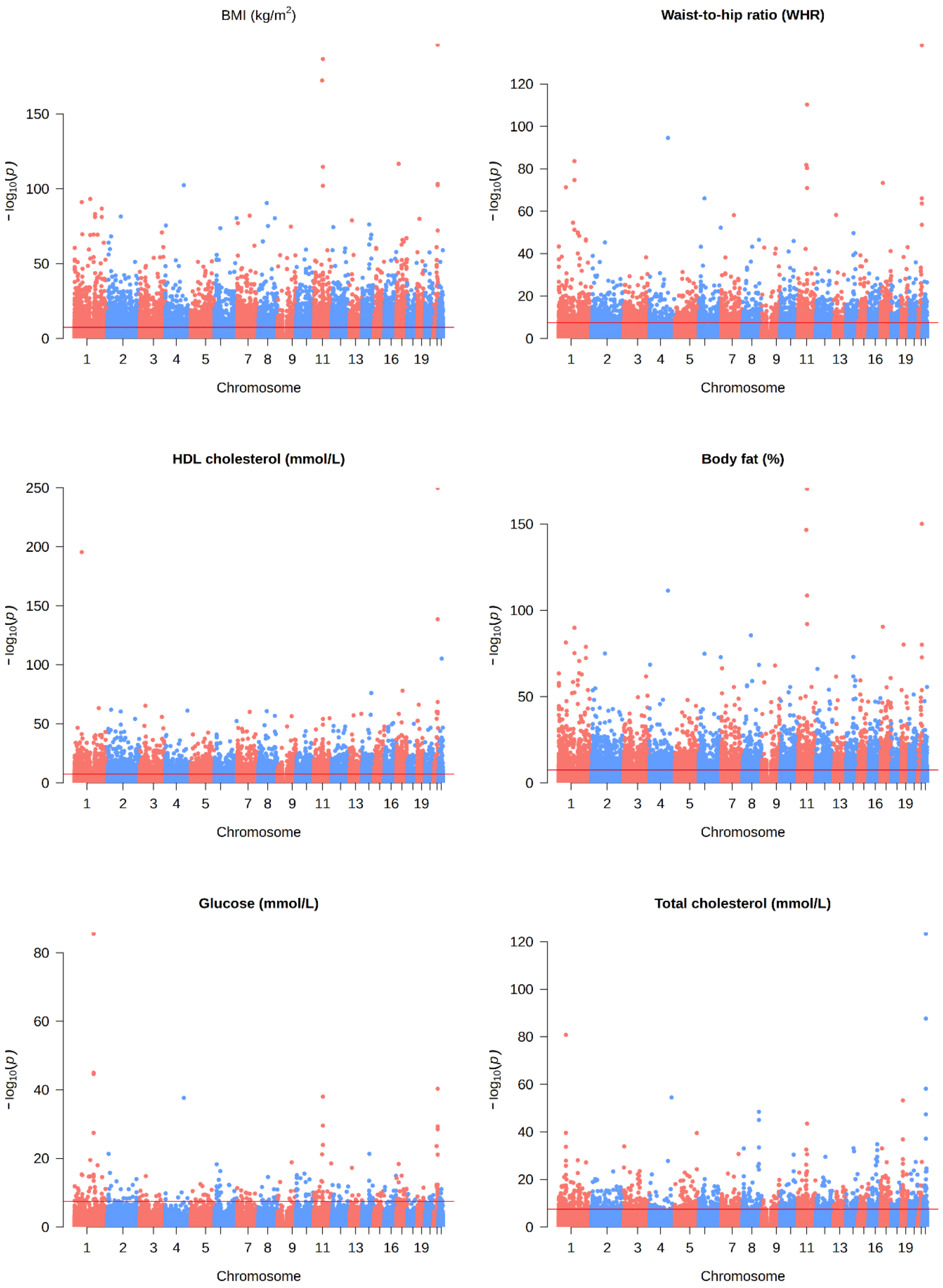

Figure S5: Manhattan plots of the results from the DNAm-PC-adjusted marginal linear regression epigenome-wide association studies of six metabolic traits in Generation Scotland. The Manhattan plots for each of the six metabolic traits show each CpG as a data point. Outcomes in each EWAS are the residuals from metabolic traits regressed on age, age^2, sex and family structure. Original outcome units are indicated in the plot titles. The x-axis shows the chromosome position, and the y-axis shows the association significance (-log$_{10}$(P)) for each CpG site. The horizontal red line indicates the significance threshold ($P < 3.6 \times 10^{-8}$). BMI =

body mass index; WHR = waist-hip ratio; HDL cholesterol = high-density lipoprotein cholesterol; DNAm = DNA methylation; PC = principal component.

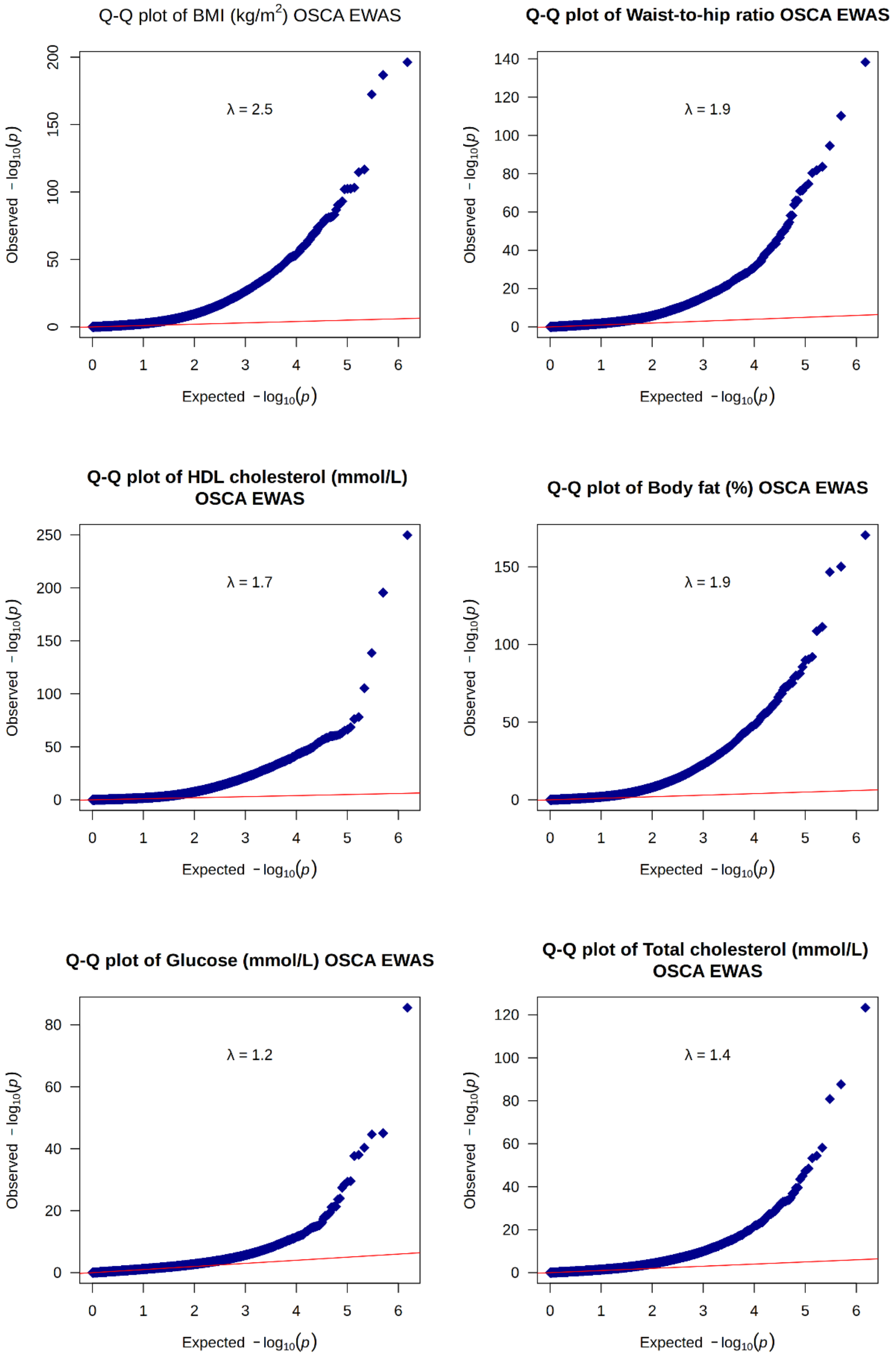

Figure S6: Quantile-Quantile plots of the results from the DNAm-PC-adjusted marginal linear regression epigenome-wide association studies of six metabolic traits in Generation Scotland. The plots show

expected –$\log_{10}$(P) by the observed –$\log_{10}$(P) for each metabolic trait. Outcomes in each EWAS are the residuals from metabolic traits regressed on age, age^2, sex and family structure. Original outcome units are indicated in the plot titles The red line shows a trend line of where the observed and expected values are the same. The inflation factor, lambda (λ), is indicated on each plot. BMI = body mass index; WHR = waist-hip ratio; HDL cholesterol = high-density lipoprotein cholesterol; DNAm = DNA methylation; PC = principal component.

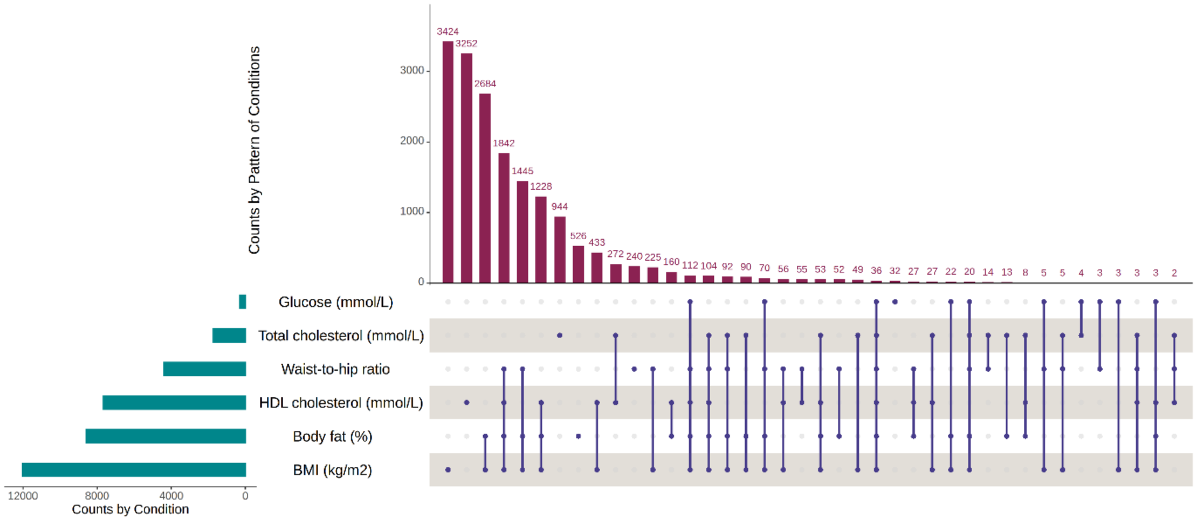

Figure S7: An upset plot of significant CpGs for the six metabolic traits in Generation Scotland. The figure shows the number of unique and overlapping CpGs for the six metabolic traits from the DNAm-PC-adjusted marginal linear regression models. Outcomes in each EWAS are the residuals from metabolic traits regressed on age, age^2, sex and family structure. Original outcome units are indicated in the plot. BMI = body mass index; WHR = waist-hip ratio; HDL cholesterol = high-density lipoprotein cholesterol; DNAm = DNA methylation; PC = principal component.

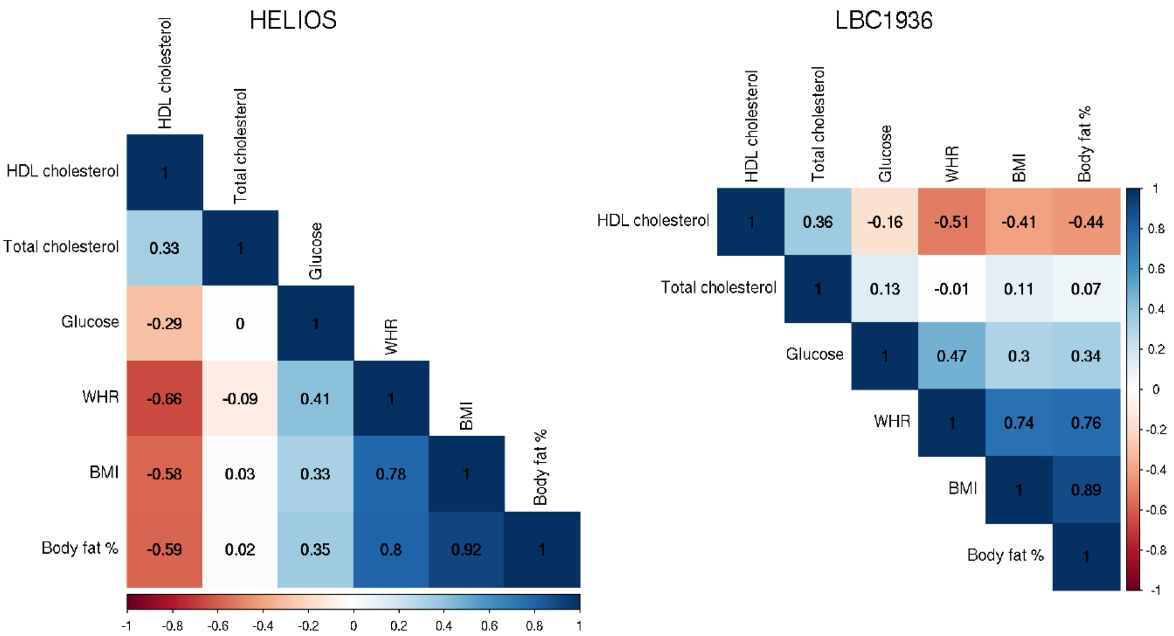

Figure S8: Metabolic EpiScore correlations for the Health for Life in Singapore (HELIOS) study and the Lothian Birth Cohort 1936 (LBC1936). The heatmaps show the Pearson correlation between each metabolic EpiScore in HELIOS and LBC1936. BMI = body mass index; WHR = waist-hip ratio; HDL cholesterol = high-density lipoprotein cholesterol.

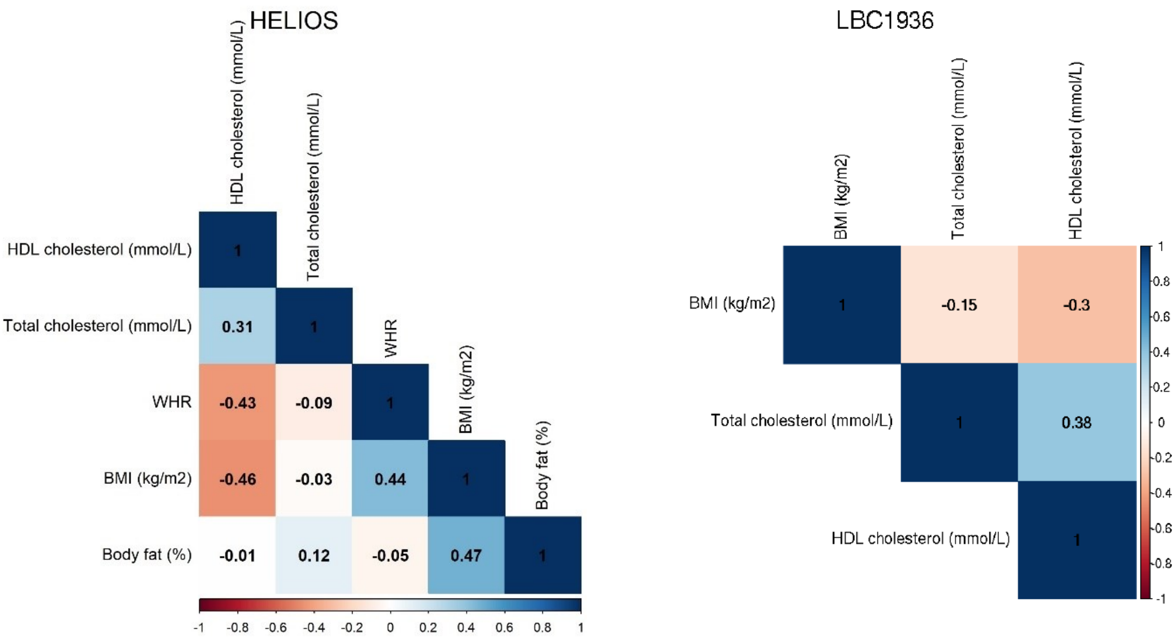

Figure S9: Metabolic trait correlations in the Health for Life in Singapore (HELIOS) study and the Lothian Birth Cohort 1936 (LBC1936). The heatmaps show the Pearson correlation between measured metabolic traits in HELIOS and LBC1936. BMI = body mass index; WHR = waist-hip ratio; HDL cholesterol = high-density lipoprotein cholesterol.

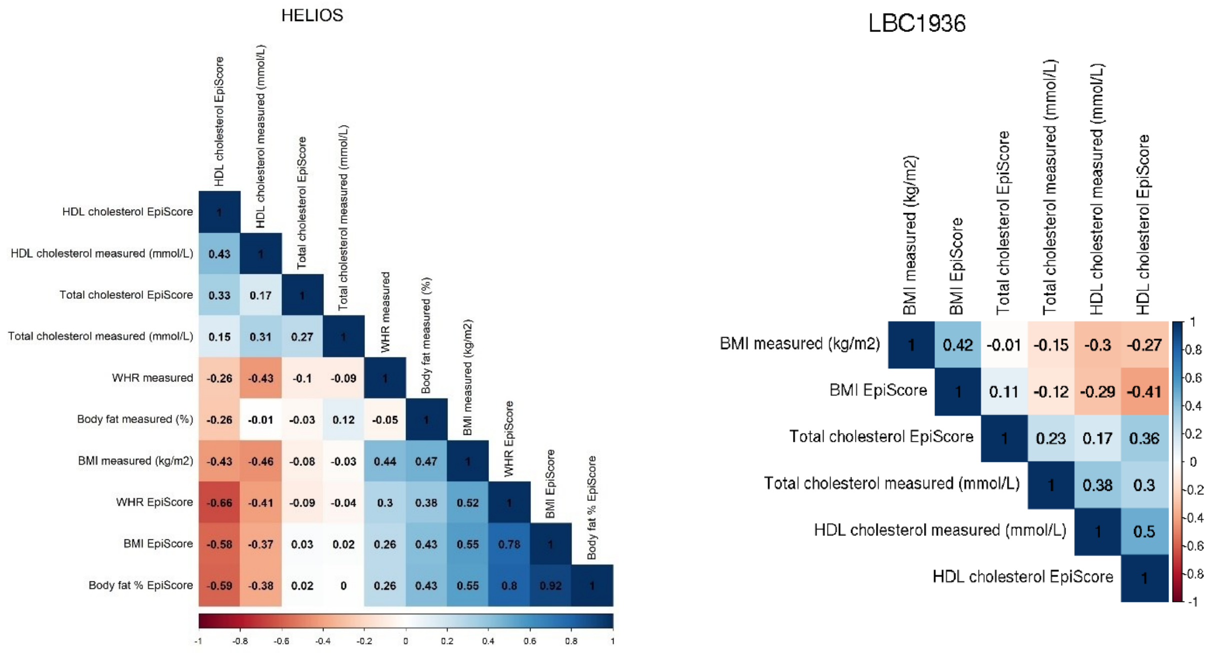

Figure S10: EpiScore-metabolic trait correlations in the Health for Life in Singapore (HELIOS) and the Lothian Birth Cohort (LBC1936). The heatmaps show the Pearson correlations between metabolic EpiScores and measured metabolic traits in HELIOS and LBC1936. BMI = body mass index; WHR = waist-hip ratio; HDL cholesterol = high-density lipoprotein cholesterol.

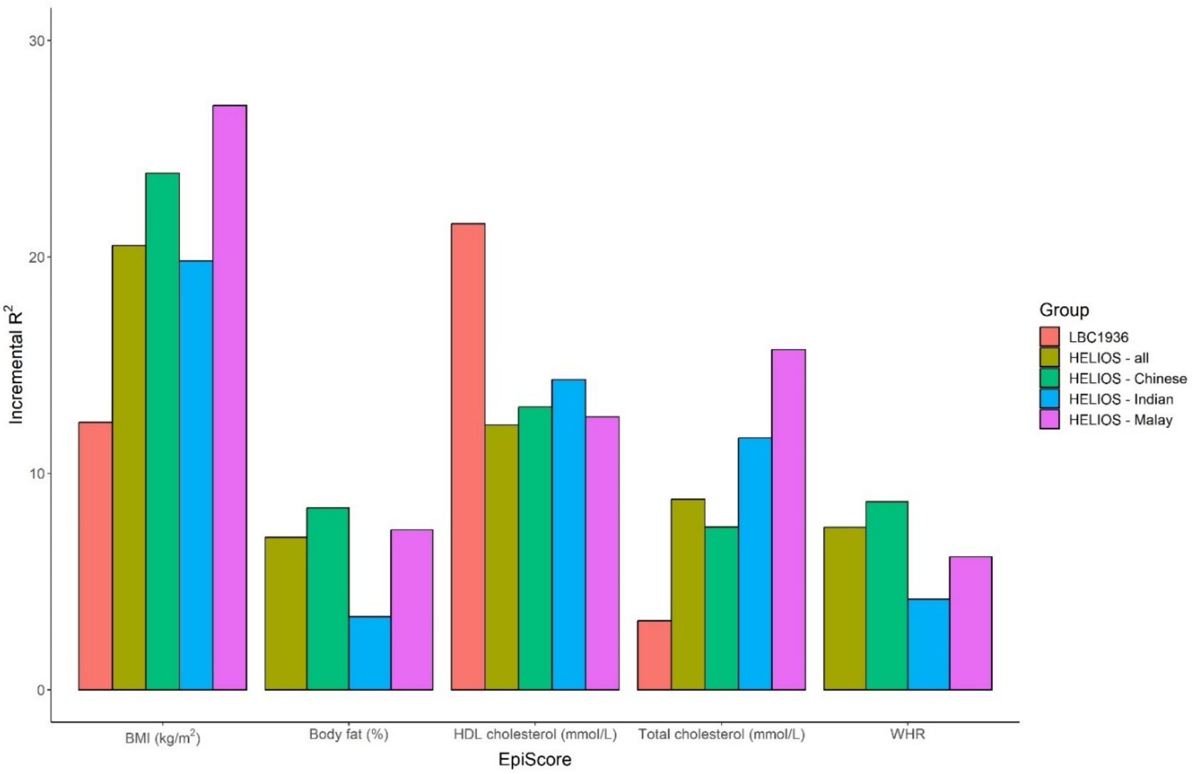

Figure S11: The variance explained in measured metabolic traits by Bayesian EpiScores in the Health for Life in Singapore (HELIOS) study and the Lothian Birth Cohort 1936 (LBC1936). The figure shows the incremental R^2 for each metabolic trait (BMI in kg/m^2; HDL cholesterol and total cholesterol in mmol/L; body fat in percentage; WHR) accounted for by their corresponding Bayesian metabolic EpiScores over and above age and sex-adjusted linear regression models in LBC1936 and HELIOS. The incremental R^2 was calculated for each subset and in the whole cohort for the HELIOS study. Full cohort models in HELIOS were additionally adjusted for subgroup (Chinese, Malay and Indian). BMI = body mass index; WHR = waist-hip ratio; HDL cholesterol = high-density lipoprotein cholesterol.

.

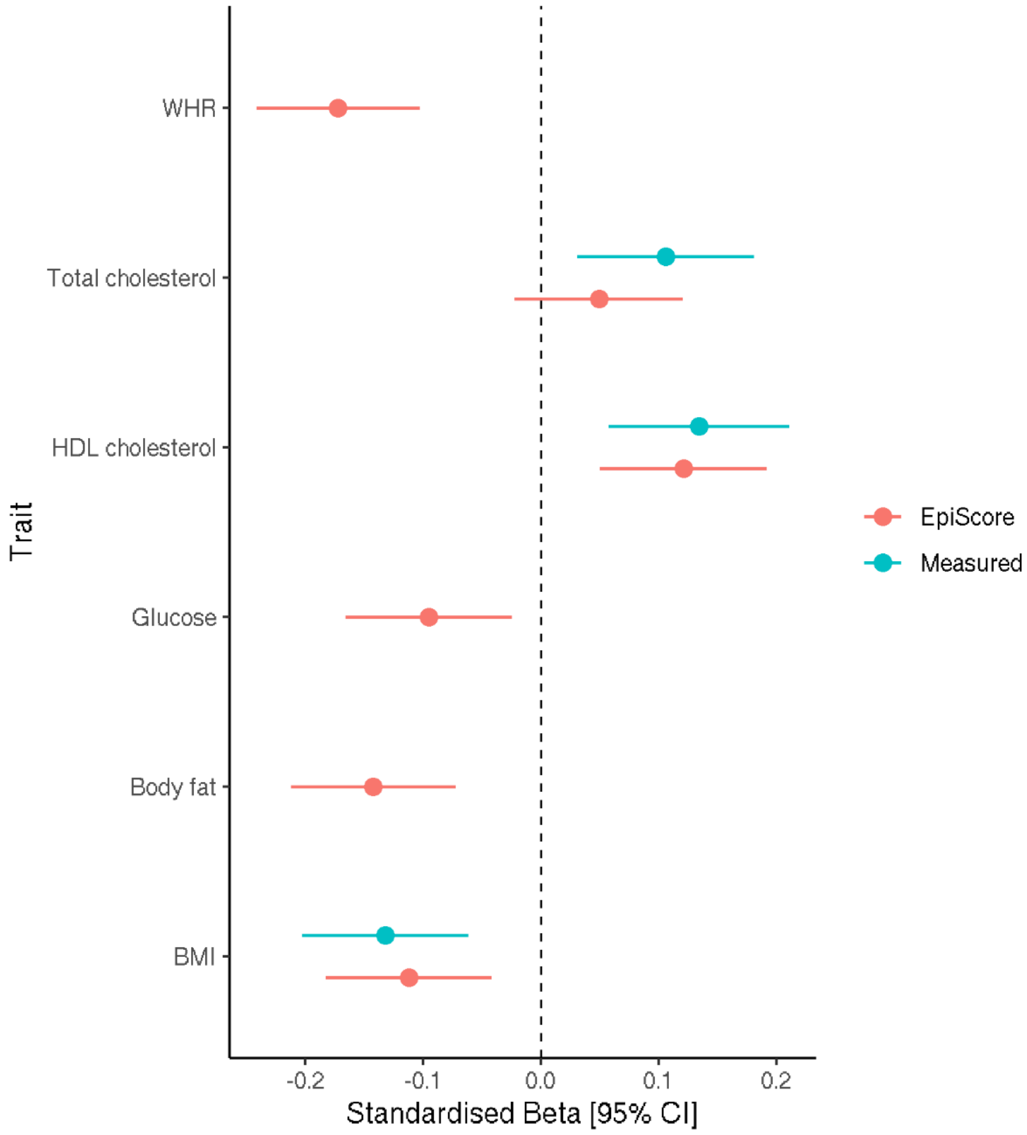

Figure S12: EpiScore and measured metabolic trait associations with general cognitive function level in the Lothian Birth Cohort (LBC1936). The figure shows associations between measured metabolic traits (BMI in kg/m^2; HDL cholesterol and total cholesterol in mmol/L) or EpiScores with general cognitive function level in models with basic adjustments (age and sex). Standardised betas are shown and error bars represent 95% confidence intervals. BMI = body mass index; WHR = waist-hip ratio; HDL cholesterol = high-density lipoprotein cholesterol.

Supplemental Methods

Metabolic measures in Generation Scotland (GS), Lothian Birth Cohort 1936 (LBC1936) and the Health for Life in Singapore (HELIOS) study

This study investigated six metabolic measures including body mass index (BMI), waist-hip ratio (WHR), body fat percentage, high-density lipoprotein (HDL) cholesterol, total cholesterol and glucose. The Generation Scotland (GS) study had all six traits available for analysis. The HELIOS study had all traits available for analysis except glucose. The LBC1936 had only BMI, HDL cholesterol and total cholesterol available for analysis. Outlier removal strategies were chosen on a cohort-by-cohort basis in line with previous approaches. In GS, BMI, body fat percentage and WHR outliers were removed by visual inspection after bivariate plots of all pairwise combinations ($n_{removed}$ = 426). Outliers > 4 standard deviations from the mean were removed for glucose, HDL cholesterol and total cholesterol ($n_{removed}$ = 173, 26, 15, respectively). In LBC1936, measured metabolic trait data were visually inspected and no outliers were removed. In HELIOS, data points were considered outliers if they were beyond 3.5 standard deviations from the mean. BMI (weight in kg /height in m^2), WHR (waist/hip circumference) and body fat percentage were measured in the clinic. In HELIOS, whole-body DEXA scans were used to quantify body fat [1]. In GS, body fat percentage is quantified with bioimpedance. HDL cholesterol, total cholesterol and glucose from blood samples were measured in mmol/L.

Bayesian EWAS

BayesR+ is a software implemented in C++ for performing Bayesian penalised regression on complex continuous traits [2]. A prior distribution is assumed as a mixture of Gaussian distributions, which correspond to groups of probes with different effect sizes. A discrete spike at zero is included, which removes probes that have a negligible effect on the trait. Informed by data from a previous analysis of BMI, prior mixture variances of probes were set to 0.0001, 0.001, 0.01 [2]. Pre-

corrected phenotype and DNA methylation data were scaled to mean zero and unit variance. Gibbs sampling was used to sample over the posterior distribution and consisted of 10,000 samples with 5,000 as burn-in. A thinning of 5 samples was applied to reduce autocorrelation. Four chains were used and the final 250 samples per chain (after thinning) were combined to form the final set of 1,000 iterations from which variance and effect size estimates were obtained. Probes with a posterior inclusion probability (PIP) ≥ 95% were deemed to be significant.

Cognitive measures in the Lothian Birth Cohort 1936 (LBC1936)

Cognitive measures in the LBC1936 have been described previously [3-6]. Cognitive testing was repeated for 5 waves at ages 70, 73, 76, 79, and 82. Thirteen cognitive measures for all five waves were available. Several cognitive domains were measured including visuospatial ability (tests: Block Design, Matrix Reasoning (WAIS-III^{UK}) and Spatial span (WMS-III^{UK})), memory (tests: Verbal Paired Associates, Logical Memory – a combination of immediate and delayed memory (WMS-III^{UK}) and Digit-span backwards (WAIS-III^{UK})), and verbal ability (tests: National Adult Reading Test, Wechsler Adult Reading Test and Verbal Fluency Test (using letters V, F and L)). Processing speed was evaluated using the Digit-symbol, Symbol Search (WAIS-III^{UK}), Choice Reaction Time and Inspection Data availability and descriptive statistics for each measure can be found in **Table S3**.

General cognitive function level and change in LBC1936

Latent measures of general cognitive function and change were generated using confirmatory factor analysis in a structural equation modelling (SEM) framework using the *Lavaan* (version 0.6-12) R package [7]. Intercepts and slopes of each cognitive test were used to indicate a latent intercept and slope (level and change) of general

cognitive function (**Table S4**). Levels and changes in cognitive functioning were modelled with a latent growth curve model (LGCM) using a Factor of Curves specification [8]. A first-order hierarchical structure was specified, and residual covariance between tests in the same cognitive domain was included, in line with a previously established correlational structure of cognitive domains (speed, memory, verbal ability and visuospatial [9]). Residual covariance between intercept and slope for individual tests was also modelled. Within-wave residual covariance between the National Adult Reading Test and the Wechsler Adult Reading Test were modelled as these tests were highly correlated. The marker method was used to scale according to the first variable to aid model convergence. Negative latent residual variances were fixed to zero. Full information maximum likelihood was used to include all data available. Confirmatory factor index (CFI), Tucker-Lewis index (TLI), root mean squared error approximation (RMSEA) and the standardised root mean squared residual (SRMR) fit measures are reported (**Table S5**). Linear regression models were run in Lavaan to test associations between general cognitive function level and change, and the metabolic traits/EpiScores with basic- and full-adjustments as follows:

Basic model: Latent G factor (intercept or slope) ~ measured trait/EpiScore + Age at baseline + Sex

Full model: Latent G factor (intercept or slope) ~ measured trait/EpiScore + Age at baseline + Sex + Scottish Index of Multiple Deprivation (SIMD) + Epigenetic smoking score (EpiSmokEr) + Alcohol units per week

Descriptive statistics for each covariate in LBC1936 can be found in **Table S6**.

Acknowledgements

This research was funded in whole, or in part, by the Wellcome Trust (218493/Z/19/Z, 104036/Z/14/Z, 108890/Z/15/Z, and 221890/Z/20/Z). For the purpose of open access, the author has applied a CC BY public copyright license to any Author Accepted Manuscript version arising from this submission. GS received core support from the Chief Scientist Office of the Scottish Government Health Directorates (CZD/16/6) and the Scottish Funding Council (HR03006). DNA methylation profiling of the GS samples was carried out by the Genetics Core Laboratory at the Edinburgh Clinical Research Facility, Edinburgh, Scotland, and was funded by the Medical Research Council UK and Wellcome (Wellcome Trust Strategic Award STratifying Resilience and Depression Longitudinally (STRADL; Reference 104036/Z/14/Z). DNA methylation data for Generation Scotland was also funded by a 2018 NARSAD Young Investigator Grant from the Brain & Behavior Research Foundation (Ref: 27404; awardee: Dr David M Howard) and by a John, Margaret, Alfred and Stewart Sim Fellowship from the Royal College of Physicians of Edinburgh (Awardee: Dr Heather C Whalley). This work was supported by the European Union Horizon 2020 (PHC.03.15, project No 666881), SVDs@Target, the Fondation Leducq Transatlantic Network of Excellence for the Study of Perivascular Spaces in Small Vessel Disease [ref no. 16 CVD 05]. We thank the LBC1936 participants and team members who contributed to these studies. The LBC1936 is supported by the Biotechnology and Biological Sciences Research Council, and the Economic and Social Research Council [BB/W008793/1] (which supports S.E.H., J.C. and A.T.), Age UK (Disconnected Mind project), the Milton Damerel Trust, the Medical Research Council (G0701120, G1001245, MR/M013111/1, MR/R024065/1) and the University of Edinburgh. Methylation typing of LBC1936 was supported by the Centre for Cognitive Ageing and Cognitive Epidemiology (Pilot Fund award), Age UK, The Wellcome Trust Institutional Strategic

Support Fund, The University of Edinburgh, and The University of Queensland. H.M.S and D.A.G are supported by funding from the Wellcome Trust 4 year PhD in Translational Neuroscience: training the next generation of basic neuroscientists to embrace clinical research [218493/Z/19/Z,108890/Z/15/Z]. S.R.C. was supported by a National Institutes of Health (NIH) research grant R01AG054628 and is supported by a Sir Henry Dale Fellowship jointly funded by the Wellcome Trust and the Royal Society (Grant Number 221890/Z/20/Z). D.L.Mc.C. and R.E.M. are supported by Alzheimers Research UK major project grant ARUK/PG2017B/10. E.B and R.E.M. are supported by Alzheimer's Society major project grant AS-PG-19b-010. R.F.H is supported by an MRC IEU Fellowship. The HELIOS study is supported by Singapore Ministry of Health's (MOH) National Medical Research Council (NMRC) under its OF-LCG funding scheme (MOH-000271-00), Singapore Translational Research (StaR) funding scheme (NMRC/StaR/0028/2017), the National Research Foundation, Singapore through the Singapore MOH NMRC and the Precision Health Research, Singapore (PRECISE) under the National Precision Medicine programme (NMRC/PRECISE/2020) and intramural funding from Nanyang Technological University, Lee Kong Chian School of Medicine and the National Healthcare Group.

1. Mina, T., Yew, Y.W., Ng, H.K., Sadhu, N., Wansaicheong, G., Dalan, R., Low, D.Y.W., Lam, B.C.C., Riboli, E., Lee, E.S. *et al.* (2023). Adiposity impacts cognitive function in Asian populations: an epidemiological and Mendelian Randomization study. The Lancet Regional Health – Western Pacific 33.
2. Trejo Banos, D., McCartney, D.L., Patxot, M., Anchieri, L., Battram, T., Christiansen, C., Costeira, R., Walker, R.M., Morris, S.W., Campbell, A. *et al.* (2020). Bayesian reassessment of the epigenetic architecture of complex traits. Nature Communications 11, 2865.
3. Deary, I.J., Gow, A.J., Pattie, A. & Starr, J.M. (2012). Cohort profile: the Lothian Birth Cohorts of 1921 and 1936. Int J Epidemiol 41, 1576-84.
4. Taylor, A.M., Pattie, A. & Deary, I.J. (2018). Cohort Profile Update: The Lothian Birth Cohorts of 1921 and 1936. International Journal of Epidemiology 47, 1042-1042r.
5. Deary, I.J., Gow, A.J., Taylor, M.D., Corley, J., Brett, C., Wilson, V., Campbell, H., Whalley, L.J., Visscher, P.M., Porteous, D.J. *et al.* (2007). The Lothian Birth Cohort 1936: a study to

examine influences on cognitive ageing from age 11 to age 70 and beyond. BMC Geriatrics 7, 28.
6. Deary, I.J., Whiteman, M.C., Starr, J.M., Whalley, L.J. & Fox, H.C. (2004). The impact of childhood intelligence on later life: following up the Scottish mental surveys of 1932 and 1947. J Pers Soc Psychol 86, 130-47.
7. Rosseel, Y. (2012). lavaan: An R Package for Structural Equation Modeling. Journal of Statistical Software 48, 1 - 36.
8. McArdle, J.J. Dynamic but Structural Equation Modeling of Repeated Measures Data. in *Handbook of Multivariate Experimental Psychology* (eds. Nesselroade, J.R. & Cattell, R.B.) 561-614 (Springer US, Boston, MA, 1988).
9. Tucker-Drob, E.M., Briley, D.A., Starr, J.M. & Deary, I.J. (2014). Structure and correlates of cognitive aging in a narrow age cohort. Psychology and Aging 29, 236-249.
