# Supplementary material for: DNA methylation-based predictors of metabolic traits in Scottish and Singaporean cohorts
Source: Am J Hum Genet. 2024 Dec 19;112(1):106–15. doi: 10.1016/j.ajhg.2024.11.012 (PMC11739919; doi:10.1016/j.ajhg.2024.11.012)
Supplement: Document S2. Article plus supplemental information [file mmc3.pdf]

# DNA methylation-based predictors of metabolic traits in Scottish and Singaporean cohorts

## Graphical abstract

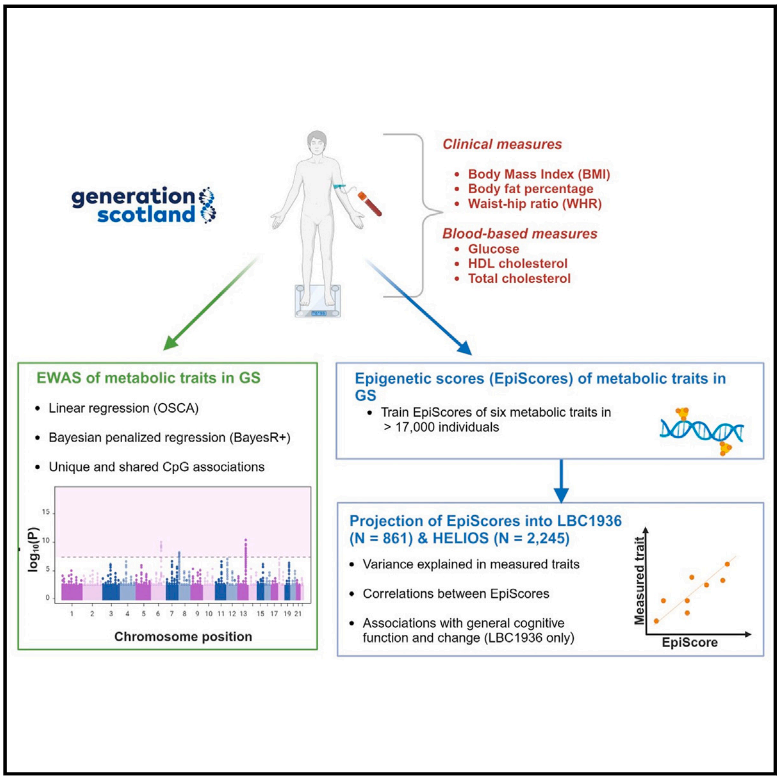



## Authors

Hannah M. Smith, Hong Kiat Ng, Joanna E. Moodie, ..., Simon R. Cox, Riccardo E. Marioni, Robert F. Hillary

## Correspondence

riccardo.marioni@ed.ac.uk



**Smith et al. performed epigenome-wide association studies (EWASs) and trained epigenetic scores (EpiScores) of six metabolic traits in >17,000 individuals from Generation Scotland. Different statistical methodologies affected the number of lead findings in the EWAS. Metabolic EpiScores were broadly applicable across diverse populations and reflected individual differences in cognitive function.**



Smith et al., 2025, The American Journal of Human Genetics *112*, 106–115
January 2, 2025

© 2024 The Author(s). Published by Elsevier Inc. on behalf of American Society of Human Genetics.

https://doi.org/10.1016/j.ajhg.2024.11.012


Check for updates

CellPress

# ARTICLE

# DNA methylation-based predictors of metabolic traits in Scottish and Singaporean cohorts

Hannah M. Smith,[1] Hong Kiat Ng,[2] Joanna E. Moodie,[3] Danni A. Gadd,[1] Daniel L. McCartney,[1] Elena Bernabeu,[1] Archie Campbell,[1] Paul Redmond,[3] Adele Taylor,[3] Danielle Page,[3] Janie Corley,[3] Sarah E. Harris,[3] Darwin Tay,[2] Ian J. Deary,[3] Kathryn L. Evans,[1] Matthew R. Robinson,[4] John C. Chambers,[2,5] Marie Loh,[2,5,6] Simon R. Cox,[3] Riccardo E. Marioni,[1,7,*] and Robert F. Hillary[1,7]

## Summary

Exploring the molecular correlates of metabolic health measures may identify their shared and unique biological processes and pathways. Molecular proxies of these traits may also provide a more objective approach to their measurement. Here, DNA methylation (DNAm) data were used in epigenome-wide association studies (EWASs) and for training epigenetic scores (EpiScores) of six metabolic traits: body mass index (BMI), body fat percentage, waist-hip ratio, and blood-based measures of glucose, high-density lipoprotein cholesterol, and total cholesterol in >17,000 volunteers from the Generation Scotland (GS) cohort. We observed a maximum of 12,033 significant findings ($p < 3.6 \times 10^{-8}$) for BMI in a marginal linear regression EWAS. By contrast, a joint and conditional Bayesian penalized regression approach yielded 27 high-confidence associations with BMI. EpiScores trained in GS performed well in both Scottish and Singaporean test cohorts (Lothian Birth Cohort 1936 [LBC1936] and Health for Life in Singapore [HELIOS]). The EpiScores for BMI and total cholesterol performed best in HELIOS, explaining 20.8% and 7.1% of the variance in the measured traits, respectively. The corresponding results in LBC1936 were 14.4% and 3.2%, respectively. Differences were observed in HELIOS for body fat, where the EpiScore explained ~9% of the variance in Chinese and Malay -subgroups but ~3% in the Indian subgroup. The EpiScores also correlated with cognitive function in LBC1936 (standardized $\beta_{range}$: 0.08–0.12, false discovery rate $p$ [$p_{FDR}$] < 0.05). Accounting for the correlation structure across the methylome can vastly affect the number of lead findings in EWASs. The EpiScores of metabolic traits are broadly applicable across populations and can reflect differences in cognition.

## Introduction

Measures of adiposity and lipids are central to profiling metabolic health. There are several clinical measures of metabolic health, which include body mass index (BMI), body fat percentage, waist-hip ratio (WHR), blood glucose levels, high-density lipoprotein (HDL) cholesterol, and total cholesterol. These traits have routinely been linked to health-related risks including cardiovascular disease,[1–3] myocardial infarction,[4] and stroke.[2,3,5] Multiple associations between metabolic traits and cognitive function and rate of cognitive decline have also been observed.[6–12] BMI is a widely assessed indicator of metabolic health but is limited by its inability to directly track the amount or distribution of fat in the body.[13,14] BMI has previously shown low specificity in identifying individuals with excess body fat.[15] Considering multiple measures that track different aspects of adiposity (and related traits) may provide a more complete assessment of metabolic health. Furthermore, exploring the molecular correlates of these metabolic indices may help to inform the shared and unique biological processes and pathways with which they are associated.

The epigenetic modification DNA methylation (DNAm) is dynamic and tissue/cell type specific and can be affected by genetic and environmental factors. Epigenome-wide association studies (EWASs) have detailed associations between individual blood-based DNAm loci (CpG sites) and metabolic traits including BMI, WHR, HDL cholesterol, and total cholesterol.[16–33] In our previous work, penalized regression models were applied to DNAm data to develop molecular predictors for a multitude of complex traits. These epigenetic scores, or EpiScores, may augment associations with health outcomes when combined with their measured phenotypic counterparts.[34–36] For example, an EpiScore for BMI increased the amount of variance in metabolic health outcomes accounted for by measured BMI alone by an average of 3%.[37] An EpiScore for WHR was also associated with all-cause mortality in the same population of healthy older adults after adjusting for measured WHR.[34]

Here, we modeled EWASs with both marginal linear regression and Bayesian penalized regression on six metabolic traits in the Generation Scotland (GS) study ($N$ > 17,000). In the former approach, we obtained marginal estimates for each CpG, which do not take into

[1]Centre for Genomic and Experimental Medicine, Institute of Genetics and Cancer, University of Edinburgh, Edinburgh, UK; [2]Lee Kong Chian School of Medicine, Nanyang Technological University, Singapore, Singapore; [3]Lothian Birth Cohorts, Department of Psychology, University of Edinburgh, Edinburgh, UK; [4]Institute of Science and Technology Austria, Am Campus 1, 3400 Klosterneuburg, Austria; [5]Department of Epidemiology and Biostatistics, School of Public Health, Imperial College London, London, UK; [6]Genome Institute of Singapore (GIS), Agency for Science, Technology and Research (A*STAR), Singapore, Singapore
[7]These authors contributed equally
*Correspondence: riccardo.marioni@ed.ac.uk
https://doi.org/10.1016/j.ajhg.2024.11.012.
© 2024 The Author(s). Published by Elsevier Inc. on behalf of American Society of Human Genetics.
This is an open access article under the CC BY license (http://creativecommons.org/licenses/by/4.0/).

account correlations across CpGs. By contrast, the Bayesian penalized regression estimated CpG effects jointly so that the effect of each CpG was conditional on all other loci. We compared findings from the individual EWASs to determine whether the six traits showed unique or common methylomic signatures. We then trained EpiScores for the six metabolic traits in GS ($N > 17{,}000$) and projected them into two independent test cohorts—the Lothian Birth Cohort 1936 (LBC1936) and the Health for Life in Singapore (HELIOS) cohort. Finally, we tested metabolic trait EpiScore associations with general cognitive function level and change in LBC1936 ($N = 861$). Associations identified between EpiScores for metabolic traits and cognitive phenotypes could offer new opportunities to examine the relevance of metabolic health indicators to aging and cognitive and neurological health outcomes.

## Methods

### GS cohort

The GS cohort has been previously described in detail.[38] Briefly, it is a Scotland-wide, family-based study of health. In the current study, 18,411 individuals had DNAm profiled on the Illumina EPIC array from blood samples taken at the study baseline between 2006 and 2011. 59% of the cohort was female, and the mean age at baseline was 47.5 years (SD: 14.9). Six metabolic measures from GS were utilized in this study: BMI (kg/m$^2$), body fat percentage, WHR, glucose (mmol/L), serum HDL cholesterol (mmol/L), and serum total cholesterol (mmol/L) (Table 1; supplemental methods). All components of GS received ethical approval from the NHS Tayside Committee on Medical Research Ethics (REC reference no. 05/S1401/89). GS has also been granted Research Tissue Bank status by the East of Scotland Research Ethics Service (REC reference no. 20-ES-0021), providing generic ethical approval for a wide range of uses within medical research. All participants signed a broad consent form. According to the terms of consent for GS participants, access to data must be reviewed by the GS access committee. Applications should be sent to access@generationscotland.org.

### The LBC1936

The LBC1936 is a longitudinal study of aging.[39,40] The study consists of individuals born in 1936, most of whom sat a general cognitive ability test at a mean age of 11 years in Scotland. Individuals living in the Lothian area were recruited to the LBC1936 study at around age 70 (baseline $N = 1{,}091$). The volunteers undertook triennial testing across five waves of follow-up (ages: ~70, 73, 76, 79, and 82). Of those with blood-based DNAm data (profiled on the Illumina 450k array) at wave 1, the mean age was 69.6 years (SD: 0.8), with 49.4% females. Three metabolic measures were utilized in this study: BMI (kg/m$^2$), serum HDL cholesterol (mmol/L), and serum total cholesterol (mmol/L) (Table 1; supplemental methods). Thirteen cognitive tests were assessed longitudinally (details in supplemental methods). Ethical approval for the LBC1936 study was obtained from the Multi-Centre Research Ethics Committee for Scotland (Wave 1, MREC/01/0/56) and the Lothian Research Ethics Committee (wave 1, LREC/2003/2/29) and the Scotland A Research Ethics Committee (waves 2–5, 07/MRE00/58). All participants provided written informed consent. These studies were performed in accordance with the Helsinki declaration. LBC data are available upon request from the LBC Study, University of Edinburgh (https://www.ed.ac.uk/lothian-birth-cohorts/data-access-collaboration). LBC data are not publicly available due to them containing information that could compromise participant consent and confidentiality.

### The HELIOS cohort

The HELIOS study is a single-center, multi-ancestry cohort of approximately 10,000 individuals residing in Singapore. A subset of the cohort in which Illumina EPIC DNAm data have been profiled has a mean age of 54.3 (SD: 11.7), and 61.2% of the cohort was female. The subset is made up of three self-reported subgroups: Chinese and other East Asian (Chinese) ($n = 1{,}778$), Malay and other South East Asian (Malay) ($n = 242$), and South Asian (Indian and other countries from the Indian subcontinent) ($n = 225$). The participants answered the following question: "what is the race as indicated in your National Identification Card?" Here, we considered three responses—Chinese, Malay, and Indian—which were used to stratify the cohort into subgroups for downstream analyses. However, we emphasize that these population descriptors may represent cultural as opposed to genetic diversity. Five metabolic measures were utilized in this study: BMI (kg/m$^2$), body fat percentage, WHR, serum HDL cholesterol (mmol/L), and serum total cholesterol (mmol/L) (Table 1; supplemental methods). The HELIOS study was approved by the National Technological University (NTU) Institutional Review Board (IRB-2016-11-030), with written informed consent obtained from each participant before the commencement of the study. HELIOS data are available upon request from the study's principal investigators. Data access requests for this study should be directed to helios_science@ntu.edu.sg.

### DNAm in GS

The GS cohort consists of 3 sets of participants, $n_{\text{set 1}} = 5{,}087$, $n_{\text{set 2}} = 4{,}450$, and $n_{\text{set 3}} = 8{,}876$, and 121 experimental batches. The Illumina Methylation EPIC (850K) array was used to quantify DNAm from whole-blood samples. A subset of set 1 included related individuals determined by family ID. Participants in set 2 were not related to each other or related to the individuals in set 1. Set 3 included some participants that were related to each other or related to participants in set 1/2. Details of the quality control (QC) performed have been published previously.[41] The QC was performed slightly differently for set 1 than sets 2 and 3. In set 1, samples were removed if $\geq 1\%$ of probes had a detection $p$ value $>0.05$ and/or the methylation-predicted sex did not match the reported sex. Further, in set 1, probes were removed if they had a bead count $<3$ or a detection $p$ value $>0.05$ in $\geq 5\%$ of individuals. In sets 2 and 3, samples were removed if $\geq 0.5\%$ of probes had a detection $p$ value $>0.01$ and/or the methylation-predicted sex did not match the reported sex. Further, in sets 2 and 3, probes were removed if they had a bead count $<3$ in $\geq 5\%$ of individuals or a detection $p$ value $>0.01$ in $\geq 1\%$ of individuals. Probes that resided on the sex chromosomes were removed in all sets. Probes that overlay SNPs and/or possible cross-hybridizing locations were removed in all sets. 15,509 probes did not meet the bead count or detection $p$ value criteria stated above. 19,681 probes belonged to the sex chromosomes. 84,352 probes overlayed SNPs and/or resided in a probable cross-hybridizing location. The union of these probes was removed, leaving 752,722 CpGs for 18,411

**Table 1. Cohort demographics for Generation Scotland, the Lothian Birth Cohort 1936, and the Health for Life in Singapore study**

| Measure | $n$ | Mean | SD | Range |
|---|---|---|---|---|
| **Generation Scotland** | | | | |
| Age (years) | 18,411 | 47.5 | 14.9 | 17.1–98.5 |
| BMI (kg/m$^2$) | 17,304 | 26.5 | 4.7 | 17–49 |
| Body fat (%) | 17,304 | 29.8 | 9.1 | 8–50 |
| WHR | 17,304 | 0.9 | 0.1 | 0.4–1.4 |
| Glucose (mmol/L) | 17,908 | 4.7 | 0.6 | 1.3–9.2 |
| HDL cholesterol (mmol/L) | 18,225 | 1.5 | 0.4 | 0.4–3.1 |
| Total cholesterol (mmol/L) | 18,270 | 5.1 | 1.1 | 0.9–9.3 |
| **Lothian Birth Cohort 1936** | | | | |
| Age (years) | 861 | 69.6 | 0.8 | 67.7–70.4 |
| BMI (kg/m$^2$) | 860 | 27.8 | 4.3 | 16–47.3 |
| HDL cholesterol (mmol/L) | 779 | 1.5 | 0.4 | 0.5–3.8 |
| Total cholesterol (mmol/L) | 851 | 5.4 | 1.2 | 2.7–10.8 |
| **Health for Life in Singapore** | | | | |
| Age (years) | 2,245 | 54.3 | 11.7 | 30.2–85.4 |
| BMI (kg/m$^2$) | 2,226 | 24.1 | 1.2 | 14.2–43.7 |
| Body fat (%) | 2,063 | 38.2 | 7.2 | 17.6–63.1 |
| WHR | 2,233 | 0.9 | 0.1 | 0.67–1.1 |
| HDL cholesterol (mmol/L) | 2,227 | 1.5 | 0.4 | 0.7–3 |
| Total cholesterol (mmol/L) | 2,223 | 5.3 | 1 | 2.4–8.6 |

A summary of the data included in this study, including $n$, mean, range, and standard deviation (SD) for each variable after outlier removal.

individuals available for analysis. Dasen normalization was carried out across all individuals.[42]

## DNAm in the LBC1936

In the LBCs of 1921 and 1936, DNAm was measured in whole-blood samples using the Illumina methylation array (450K) in three sets: $n_{\text{set }1} = 2{,}195$, $n_{\text{set }2} = 996$, and $n_{\text{set }3} = 552$. The QC of the data has been described previously.[43] Duplicate samples were run to help quantify batch effects. The poorest-performing duplicates were removed during QC. Samples and probes with low call rates (call rate $\geq$95% at $p$ value <0.01) were removed. Probes that resided on the sex chromosomes were removed. The dataset was subset to the LBC1936 data and set 1 only. 459,310 CpGs for 861 individuals were available for analysis after QC. Beta values were background corrected and normalized to controls using the minfi packaged in R.[44]

## DNAm in HELIOS

DNAm from whole-blood samples in the HELIOS cohort was measured using the Illumina HumanMethylation EPIC array after bisulfite conversion of DNA was carried out according to the manufacturer's protocol (EZ DNA Methylation Kit). The minfi software package[44] was used to obtain bead intensity, with a detection rate of $p < 0.02$ used for marker calling. Probes with call rates <95% were excluded. Samples were excluded for array scanning failures ($n = 2$), if the methylation-predicted sex did not match the reported sex ($n = 39$), and duplication ($n = 17$). 2,445 samples with 837,722 CpG sites were available for analysis after QC. Quantile normalization was used to account for batch effects. The HELIOS DNAm data were processed as a whole cohort; therefore, there is no difference between probe sets across the Chinese, Malay, and Indian subgroups.

## EWASs of six metabolic traits in GS

Linear regression models tested for associations between 752,722 CpG sites and each of the six metabolic traits in GS using the fast linear method in the omics-data-based complex trait analysis (OSCA) software.[45] To facilitate less computationally expensive analyses, phenotypes were regressed on age, age$^2$, sex, and family structure (to account for relatedness in GS) using linear mixed-effects models (lmekin function from the coxme package [v.2.2.18.1, https://CRAN.R-project.org/package=coxme] in R). Family structure was modeled as a random effect via a kinship matrix constructed using the R package kinship2 (v.1.9.6, https://CRAN.R-project.org/package=kinship2). This incorporates maternal and paternal identifiers for each participant in the cohort as a matrix e.g., values of 0.5 are specified for parent-offspring or sibling pairs. CpG M-values were pre-corrected for age, sex, and experimental batch ($n = 121$ batches) in linear regression models using the lm function in R. Residuals from the regression models for each outcome trait and CpG were taken forward for the EWASs. An epigenetic smoking score, EpiSmokEr, was derived using the smoking score (SSc) method from the EpiSmokEr R package.[46] The SSc method multiplies methylation levels of 187 CpG

sites using weights from a study by Zeilinger et al. that found these sites to be significantly associated with smoking.[46,47] The multiplied methylation levels at 187 sites are then summed for each individual to calculate their smoking score. EpiSmokEr scores and Houseman-estimated white blood cell (WBC) proportions[48] were included as fixed-effect covariates in the OSCA analysis. A sensitivity analysis was carried out by additionally including the first 20 methylation-based principal components (PCs) as covariates to account for potentially unmeasured confounders. Adjustments for inflation and bias were carried out on the results from the DNAm-PC-adjusted models using the bacon package (v.1.18.0) in R.[49] Descriptive statistics can be found in Table S1. A significance level of $p < 3.6 \times 10^{-8}$ was set to detect significantly associated CpGs as suggested by Saffari et al. in a study investigating significance thresholds in EWASs using a simulation approach.[50] Mapping of CpG sites to genes was performed using the "MethylationEPIC_v-1-0_B2.csv" file from the zip archive "infiniummethylationepic-v1-0-b2-manifest-file-csv.zip" from www.illumina.com. The annotation file is in build hg19. Principal-component analyses (PCAs) were performed on the significantly associated CpG sites from each metabolic trait EWAS. The number of approximate independent signals was denoted as the cumulative number of PCs that accounted for at least 80% of the variance among all significantly associated probes. PCA was performed using the scikit-learn package in Python (2.7.17).[51]

### Bayesian EWAS

Probe-by-probe (marginal) linear regression models fail to consider the correlation structure that exists across the methylome. Therefore, we considered Bayesian penalized regression, conducted using BayesR+,[52] as a secondary analysis. This method estimates single marker or probe effects while controlling for all other probes as well as being able to control for known and unknown confounding variables. This method also estimates the amount of phenotypic variation attributed to genome-wide DNAm. We applied the same covariate and phenotype preparation strategy as in the linear regression models. Significant CpGs were defined as sites with a posterior inclusion probability (PIP) $\geq 0.95$. Details on the methods used for the Bayesian strategy can be found in the supplemental methods.

### Replication of previous literature

The EWAS Catalog[16] was used to determine if the overlapping CpGs that were found to be associated with all six metabolic traits in the DNAm-PC-adjusted linear regression EWASs have previously been identified in other studies. The EWAS Catalog was filtered to whole-blood samples, CpG-metabolic trait associations with $p < 3.6 \times 10^{-8}$ (in line with our study and consistent with Saffari et al.[50]), and study sample $n > 1{,}000$ participants. The number of studies that met our criteria and the search terms used to identify studies from the EWAS Catalog can be found in Table S2. The EWAS Catalog was filtered to exclude studies that GS contributed data toward.

### Generation and projection of DNAm-based proxies of six metabolic traits

Penalized regression models were trained in GS to generate the EpiScores of each of the six metabolic traits using the R package biglasso (v.1.5.2).[53] Each trait was modeled as the response variable (using the same phenotype files from the EWASs). DNAm is measured with the EPIC array in GS and HELIOS, while the 450K array was used in the LBC1936. Therefore, the intersection of 395,380 post-QC sites between GS and LBC1936 were considered as potential predictors. Cross-validation was carried out ($n_{\text{folds}} = 20$), and an elastic net (elnet) penalty was set (alpha = 0.5). CpG sites with a non-zero coefficient were retained and used to derive EpiScores in LBC1936 ($N = 861$). This was followed by further testing in the HELIOS cohort ($N = 2{,}245$). All three datasets (GS, LBC1936, and HELIOS) were pre-processed and normalized independently, including the mean imputation of missing CpG values. Predictors obtained from the Bayesian penalized regression models were also projected into LBC1936 and HELIOS using the mean posterior effect sizes as weights for the scores. The variance explained (incremental $R^2$) in each metabolic trait by their corresponding EpiScore over and above age and sex in linear regression models was then calculated. In HELIOS, the variance explained was calculated in the full cohort and in the Chinese, Malay, and Indian subgroups. In HELIOS full-cohort models, subgroup was additionally included as a covariate.

### EpiScore associations with general cognitive function and change in LBC1936

A latent intercept and age-related slope for general cognitive function were generated in LBC1936 using a structural equation modeling (SEM) framework with the R package Lavaan (v.0.6.12).[54] Measured traits and EpiScores were regressed on intercepts and slopes in separate linear models. Full details are provided in supplemental methods and Tables S3–S6.

## Results

### EWASs of six metabolic traits

Correlations between metabolic traits, covariates, and the first 20 DNAm PCs in GS ranged between −0.36 (WHR and HDL cholesterol) and 0.6 (BMI and body fat percentage) and are shown in Figure S1. The largest absolute correlation between the PCs and outcomes (covariates) was r = 0.09 for PC2 and BMI (and r = 0.27 for PC1 and B cells). Marginal linear regression EWASs of six metabolic traits were performed in GS, adjusting for estimated WBC proportions, and EpiSmokEr. The number of CpG sites significantly associated ($p < 3.6 \times 10^{-8}$) with each of the traits is summarized in Table 2. This ranged between 460 for glucose to 57,307 for BMI. Manhattan plots can be observed in Figure S2, and the top 1,000 significantly associated CpGs with each trait are listed in Table S7. Full summary statistic output is publicly available at Zenodo: https://doi.org/10.5281/zenodo.13998835 and Edinburgh Data Share: https://datashare.ed.ac.uk/handle/10283/8877.

The large number of significant associations observed in our models may reflect correlation structures among CpG sites (quantile-quantile [Q-Q] plots and inflation factors—which ranged between 1.8 and 7.4—can be observed in Figure S3). Therefore, we performed PCA on the significant CpGs ($p < 3.6 \times 10^{-8}$) for each trait to determine the approximate number of independent features present. We identified between 82 and 4,354 (for glucose and BMI, respectively) PCs or "independent features" that

**Table 2. The number of significantly associated CpGs with each metabolic trait in Generation Scotland**

| Trait | No. of significant CpGs | | | No. of PCs for ≥80% of variance explained in significant CpGs | |
|---|---|---|---|---|---|
| | non-PC-adjusted marginal EWAS at $p < 3.6 \times 10^{-8}$ | DNAm-PC-adjusted marginal EWAS at $p < 3.6 \times 10^{-8}$ | Bayesian EWAS at PIP ≥ 0.95 (overlap in DNAm-PC-adjusted marginal EWAS) | non-PC-adjusted marginal EWAS | DNAm-PC-adjusted marginal EWAS |
| BMI ($kg/m^2$) | 57,307 | 12,033 | 27 (25) | 4,354 | 1,309 |
| WHR | 20,622 | 4,411 | 12 (11) | 2,659 | 696 |
| Body fat (%) | 29,302 | 8,592 | 18 (17) | 3,283 | 1,198 |
| Glucose (mmol/L) | 460 | 316 | 3 (1) | 82 | 83 |
| HDL cholesterol (mmol/L) | 32,288 | 7,674 | 20 (16) | 2,734 | 1,088 |
| Total cholesterol (mmol/L) | 1,645 | 1,740 | 19 (18) | 376 | 328 |

The table shows the number of significantly associated CpGs with each metabolic trait using marginal linear regression, marginal DNAm-PC-adjusted linear regression, and Bayesian penalized regression. The table also shows the number of PCs that account for ≥80% of the variance of the significant CpGs from both of the marginal linear regression analyses for each metabolic trait. Outcomes in each EWAS are the residuals from metabolic traits regressed on age, $age^2$, sex, and family structure. Original outcome units are indicated in the table.

accounted for ≥80% of the variance in the underlying CpG sites (Table 2). Next, we performed a sensitivity analysis that further adjusted the linear regression models for the first 20 DNAm PCs. The first 20 PCs explain 21.6% of the total variance in the methylation data (Figure S4). The number of significant CpG sites ranged between 316 for glucose and 12,033 for BMI. The number of PCs that explained 80% of the variance in the significant loci for each trait ranged between 83 and 1,309 (Table 2). The top 1,000 significantly associated CpGs for each trait can be found in Table S8. Manhattan and Q-Q plots for each trait can be found in Figures S5 and S6. Given that the number of significant CpG associations was still relatively large after further adjusting for 20 DNAm PCs, we corrected the effect sizes and $p$ values of the DNAm-PC-adjusted results for inflation and bias using the bacon method.[49] This resulted in between 206 (for glucose) and 4,390 (for HDL cholesterol) significant CpG associations (Table S9).

Finally, we performed Bayesian penalized regression, which jointly models all CpGs and accounts for genome-wide correlation patterns. Table 2 shows the number of high-confidence associations (PIP ≥ 0.95), which ranged between 3 (glucose) and 27 associations (BMI) (Table S10). The majority of these significant findings overlapped with those observed using the DNAm-PC-adjusted marginal linear regression approach (Table 2). Using the Bayesian method, we obtained estimates for the variance captured by genome-wide DNAm that ranged between 24% for WHR and 53% for BMI (Table S11).

36 CpG sites were significant ($p < 3.6 \times 10^{-8}$) across all six metabolic traits in the marginal linear regression models adjusted for DNAm PCs (Table S12; Figure S7). In the Bayesian models, a single CpG site, "cg06500161" (mapped to *ABCG1*), was associated with BMI, body fat percentage, HDL cholesterol, total cholesterol, and WHR (PIP ≥ 0.95; Table S10).

13 of the 36 common CpGs from the DNAm-PC-adjusted linear models had been previously associated with metabolic traits in studies using whole-blood samples at $p < 3.6 \times 10^{-8}$ and study sample $n > 1{,}000$ reported in the EWAS Catalog (Table S12). Of the 36 CpGs associated with all traits in the linear models, four mapped to *CPT1A*, four mapped to *ABCG1*, and three mapped to *PHGDH*. Seven of the overlapping CpGs did not map to any genes. The remaining 18 CpGs mapped to unique genes, giving a total of 21 unique genes containing the overlapping CpGs.

### EpiScores of metabolic traits tested in the LBC1936 and HELIOS

EpiScores for each of the six metabolic traits were trained in GS using elnet penalized regression and projected into the LBC1936 and HELIOS cohorts. We explored how much additional variance could be accounted for in each metabolic trait by the corresponding EpiScore over and above linear regression models adjusting for age and sex. In the LBC1936, EpiScores accounted for 3.2% of the variance for total cholesterol, 18.5% for HDL cholesterol, and 14.4% of the variance in BMI. In HELIOS full-cohort analysis, the incremental $R^2$ estimates ranged between 7.1% (for total cholesterol) and 20.8% (for BMI). However, there was variability within the subsets of HELIOS. Most notably, the body fat percentage EpiScore accounted for 9.2% and 9.5% in the Chinese and Malay subgroups but only 3.1% in the Indian subgroup (Figure 1; Table S13). In LBC1936 and HELIOS, the correlations between all six EpiScores are shown in Figure S8. Correlations between measured traits ranged from −0.3 to 0.38 for LBC1936 and from −0.46 to 0.47 for HELIOS (Figure S9). Correlations between measured traits and EpiScores ranged between −0.41 and 0.5 in LBC1936 and −0.66 and 0.92 in HELIOS (Figure S10).

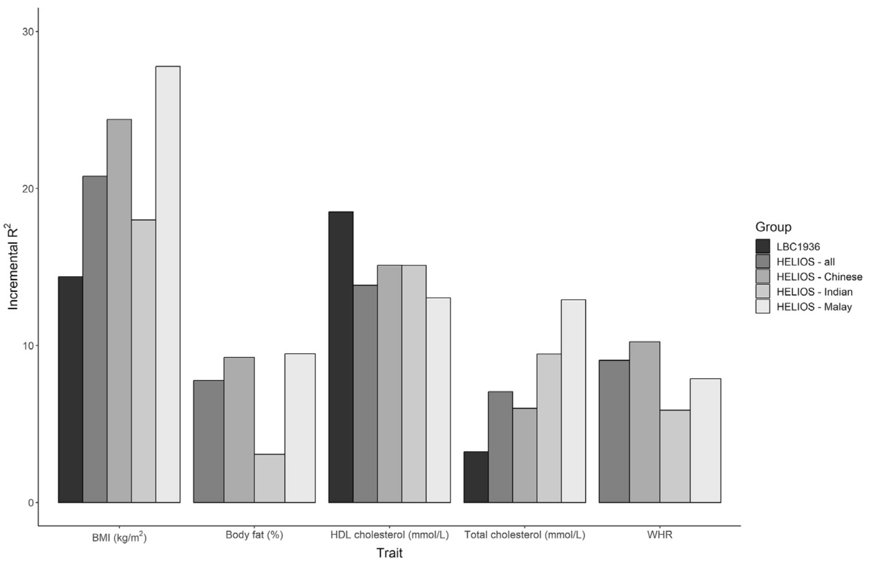


**Figure 1. The variance explained in measured metabolic traits by elnet EpiScores in the LBC1936 and the HELIOS study**

Additional variance (incremental $R^2$) accounted for in each metabolic trait (BMI in $kg/m^2$; HDL cholesterol and total cholesterol in mmol/L; body fat in percentage; WHR) by their corresponding elnet EpiScores over and above age- and sex-adjusted (and subgroup—Chinese, Malay, and Indian—in the Health for Life in Singapore [HELIOS] full cohort) linear regression models in the Lothian Birth Cohort 1936 (LBC1936) and HELIOS. Measured glucose levels were not available for either cohort. Incremental $R^2$ was calculated for each subgroup and in the whole cohort in HELIOS.

Next, we tested the Bayesian EpiScores in both LBC1936 and HELIOS, observing similar results to the elnet approach (Figure S11; Table S13).

### EpiScore associations with general cognitive function

Metabolic traits have previously been linked to cognitive outcomes. Given this, we tested if the metabolic (elnet) EpiScores were associated with general cognitive function level and longitudinal changes in the LBC1936 ($N = 861$). In models adjusting for age and sex, the three measured traits (BMI, total cholesterol, and HDL cholesterol) and all EpiScores, except the total cholesterol EpiScore, were significantly associated with general cognitive function (intercept) in LBC1936 (false discovery rate $p$ [$p_{FDR}$] $< 0.05$; Figure S12; Table S14). In fully adjusted models, significant ($p_{FDR} < 0.05$) EpiScore associations were observed for WHR, glucose, body fat percentage, and BMI (standardized $\beta_{range}$: −0.08 to −0.12) and for measured BMI (standardized β: −0.10; Figure 2A). No significant associations were observed with general cognitive change over ~12 years (mean age 70 to mean age 82) of follow-up ($p_{FDR} > 0.05$; Table S14). A combination of the EpiScore and measured trait accounted for more variance explained in general cognitive function level than an EpiScore or measured trait alone (Figure 2B; Table S15). EpiScores explained more variance than the measured trait for general cognitive function by an average of 0.3%.

## Discussion

EWASs of six metabolic traits were performed in GS ($N > 17{,}303$). A large number of significantly associated CpGs were identified for each trait via linear regression models adjusted for WBCs and EpiSmokEr (marginal associations with $p < 3.6 \times 10^{-8}$ ranged from 460 to 57,307 per trait). Further adjustments for the first 20 DNAm PCs reduced the number of significant findings (from 316 to 12,033 per trait), most likely by adjusting for poorly measured or unknown confounders. A Bayesian approach, which modeled the CpGs jointly and conditionally upon each other, resulted in between 3 and 27 high-confidence (PIP ≥ 0.95) CpG associations for the six traits. As shown in extensive simulation work,[52] BayesR+ yields a better FDR than marginal regression approaches. Whereas the lead loci identified in BayesR+ were contained in the list of significant DNAm-PC-adjusted results, they can be considered with high confidence as lead loci. EpiScores for each metabolic trait were trained in GS and projected into two independent test cohorts, LBC1936 and HELIOS. The metabolic EpiScores were tested for associations with general cognitive function level and change. Four of the EpiScores were associated with general cognitive function in fully adjusted models ($p_{FDR} < 0.05$), but none were associated with longitudinal cognitive change.

36 CpGs were associated with all six traits when using the DNAm-PC-adjusted marginal linear regression modeling approach. This included 13 CpGs previously linked to metabolic traits in the literature referenced in the EWAS Catalog.[17–25,32,55–57] However, it is worth noting that the EWAS Catalog is not extensive, and some studies may not be reported. Several genes the 36 CpGs mapped to had known metabolic functions. *ABCG1* and *ABCA1* encode two proteins that are part of the ABC transporter superfamily involved in the transport of cholesterol.[58,59] *CPT1A* encodes a rate-limiting fatty acid oxidation enzyme that oxidizes medium and long acyl-coenzyme A (CoA) esters, an important step that allows these molecules access to the inner mitochondrial membrane.[60] PDK4 is a kinase that inhibits the pyruvate dehydrogenase complex (PDC), which is responsible for the decarboxylation of pyruvate to acetyl-CoA.[61] The inhibition of PDC results in a switch from glucose oxidation to fatty acid oxidation, and PDK4 has been suggested as a marker for increased fatty acid

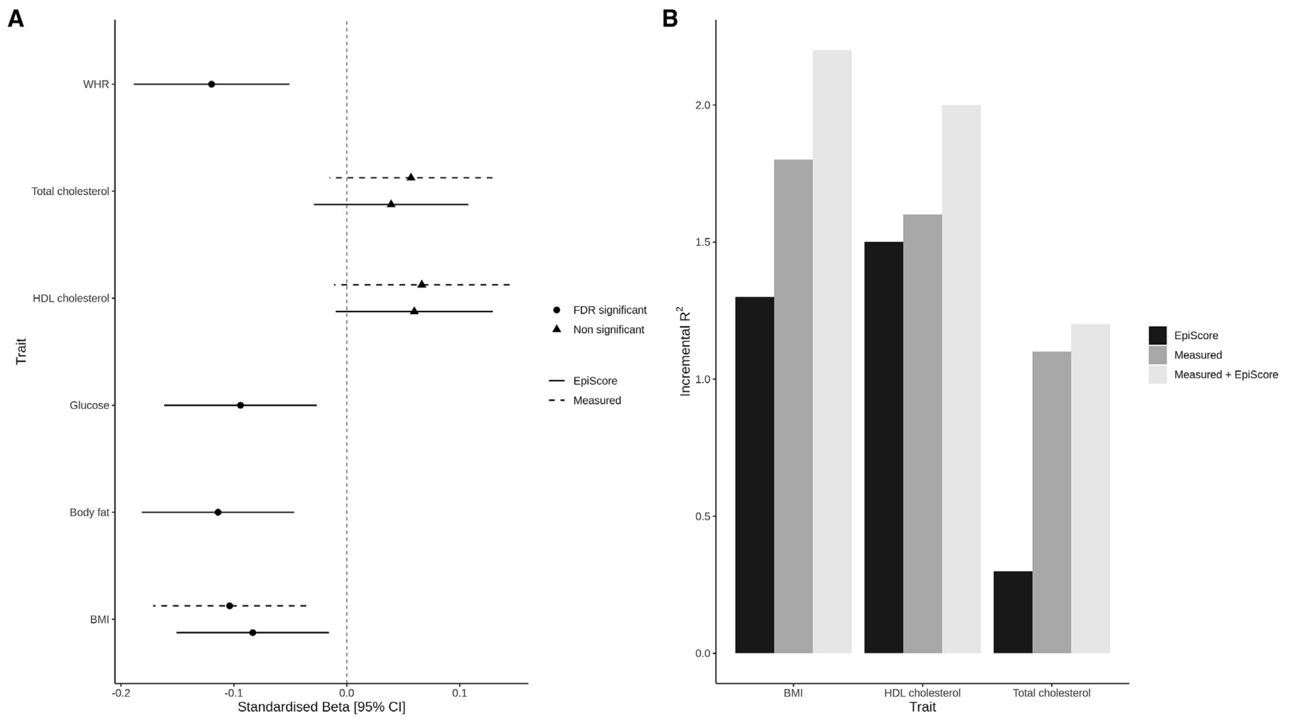


**Figure 2. EpiScore and measured metabolic traits in relation to general cognitive function level in the LBC1936**
(A) Associations between measured traits (BMI in kg/m$^2$; HDL cholesterol and total cholesterol in mmol/L) or EpiScores with general cognitive function level in models with full adjustments. Standardized betas are shown, and error bars represent 95% confidence intervals. The point estimates for associations that were significant after multiple test correction (FDR significant) are shown as circles. The point estimates for non-significant associations are shown as triangles.
(B) Additional variance accounted for in general cognitive function level by measured metabolic traits (BMI in kg/m$^2$, HDL cholesterol and total cholesterol in mmol/L), metabolic EpiScores, and both combined, over and above linear regression models adjusted for age and sex.

oxidation.[61,62] Previous studies have used Mendelian randomization approaches to suggest that DNAm is more likely to be a consequence than a cause of differences in BMI and HDL cholesterol.[23,55,63] Future work could prioritize our lead CpGs to explore casual pathways across the remaining metabolic traits.

Metabolic EpiScores accounted for additional variance in metabolic traits over and above age and sex in both LBC1936 and HELIOS. The elnet EpiScores for BMI and total cholesterol accounted for more variance in their corresponding measured traits in the HELIOS full cohort than in the LBC1936. Conversely, the EpiScore for HDL cholesterol accounted for more variance in the LBC1936 than the HELIOS full cohort. The performance of elnet metabolic EpiScores in HELIOS varied across the Chinese, Malay, and Indian subgroups. In particular, the body fat percentage EpiScore performed similarly in the Chinese and Malay subgroups (∼9% variance accounted for) but had a much lower performance in the Indian subgroup (3.1% variance accounted for). Within the Asian population, it has been reported that Indians have a higher body fat percentage compared with Chinese and Malay populations.[64] Asian Indian individuals also have been shown to have increased total and centrally distributed body fat compared with those of European ancestries.[65] Previous EWASs have found evidence for CpG-BMI associations to differ by ancestry, although sample sizes varied considerably between groups.[33] Here, a more robust comparison of EpiScores would be aided by using equivalently sized cohorts for each subpopulation and by training/testing across all subgroups—which could be defined by both genetic similarities and cultural identities. Future work should also investigate if factors such as diet or cultural environment can help to refine our understanding of why some—but not all—EpiScores perform well across different populations.

The potential usefulness of using DNAm to impute measured traits in studies where they are not available was highlighted by the similarity of effect sizes between metabolic EpiScores and their corresponding measured traits in models predicting general cognitive function levels. In the future, these EpiScores could be explored longitudinally in the LBC studies to determine if they change in tandem with their measured traits or with concurrent physical and cognitive decline.

This study has multiple strengths, including large sample sizes, the use of multiple diverse cohorts, a multimethod approach (marginal linear regression and Bayesian penalized regression), volunteers from a wide range of ages across adulthood, and longitudinal data to test for cognitive changes in late-life testing (LBC1936). Of the two EWAS strategies, and despite adjustments for relevant covariates, the marginal linear regression approach yielded

a vast number of significant CpGs associated with each metabolic trait. However, this approach is naive in that it does not account for the genome-wide correlation patterns and structure across the methylome. This leads to an inflation in the number of significant findings and biased estimation of effect sizes. Using more stringent methods like BayesR+ helped to overcome such issues, resulting in a high-confidence set of CpG-trait associations. A limitation is that only three of the six metabolic traits were measured in LBC1936; therefore, we were unable to compare EpiScore performance against measured WHR, glucose, and body fat percentage in this cohort. A further limitation lies in the methodological differences in the measuring of metabolic traits in each cohort. For example, body-fat percentage is measured via bioimpedance in GS, whereas dual energy X-ray absorptiometry (DEXA) scans were used in the HELIOS cohort. Finally, alternative strategies for feature pre-selection prior to training EpiScores are likely to result in improved predictors.[33,66,67]

To conclude, our findings suggest that different EWAS strategies (i.e., marginal linear models and conditional Bayesian models) vastly alter the number of significant CpGs associated with metabolic traits. As increasingly large cohorts with DNAm are generated, conditional analyses will help to control false positive rates, although they will not identify all correlated/co-dependent sites under a peak. We have also shown that metabolic EpiScores trained in a Scottish population perform well in external Scottish and Singaporean cohorts. However, further testing is required in, e.g., subpopulations of different genetic and cultural backgrounds to determine how well the predictors generalize. Further, metabolic EpiScores and measured metabolic traits had comparable magnitudes of association with general cognitive function. This highlights the potential usefulness of metabolic EpiScores to impute the corresponding traits where they have not been measured in a cohort.

## Data and code availability

All code associated with this manuscript is available for open access at the following GitHub repository: https://github.com/marioni-group/Metabolic_trait.

The full EWAS summary statistic output is publicly available at Zenodo: https://doi.org/10.5281/zenodo.13998835 and Edinburgh Data Share: https://datashare.ed.ac.uk/handle/10283/8877.

## Declaration of interests

R.E.M. is an advisor to the Epigenetic Clock Development Foundation and Optima Partners, Ltd. D.A.G. and D.L.M. are employed by Optima Partners, Ltd., in a part-time capacity. R.F.H. has acted as a scientific consultant to Optima Partner, Ltd., and has received consultant fees from Illumina.

## Supplemental information

Supplemental information can be found online at https://doi.org/10.1016/j.ajhg.2024.11.012.

Received: June 11, 2024
Accepted: November 26, 2024
Published: December 19, 2024

## References

1. Khan, S.S., Ning, H., Wilkins, J.T., Allen, N., Carnethon, M., Berry, J.D., Sweis, R.N., and Lloyd-Jones, D.M. (2018). Association of Body Mass Index With Lifetime Risk of Cardiovascular Disease and Compression of Morbidity. JAMA Cardiol. *3*, 280–287.
2. Alloubani, A., Nimer, R., and Samara, R. (2021). Relationship between Hyperlipidemia, Cardiovascular Disease and Stroke: A Systematic Review. Curr. Cardiol. Rev. *17*, e051121189015.
3. Salinero-Fort, M.A., Andrés-Rebollo, F.J.S., Cárdenas-Valladolid, J., Méndez-Bailón, M., Chico-Moraleja, R.M., de Santa Pau, E.C., Jiménez-Trujillo, I., Gómez-Campelo, I., de Burgos Lunar, C., de Miguel-Yanes, J.M.; and MADIABETES (2021). Cardiovascular risk factors associated with acute myocardial infarction and stroke in the MADIABETES cohort. Sci. Rep. *11*, 15245.
4. Cao, Q., Yu, S., Xiong, W., Li, Y., Li, H., Li, J., and Li, F. (2018). Waist-hip ratio as a predictor of myocardial infarction risk: A systematic review and meta-analysis. Medicine (Baltim.) *97*, e11639.
5. Wang, X., Dong, Y., Qi, X., Huang, C., and Hou, L. (2013). Cholesterol levels and risk of hemorrhagic stroke: a systematic review and meta-analysis. Stroke *44*, 1833–1839.
6. Karlsson, I.K., Gatz, M., Arpawong, T.E., Dahl Aslan, A.K., and Reynolds, C.A. (2021). The dynamic association between body mass index and cognition from midlife through late-life, and the effect of sex and genetic influences. Sci. Rep. *11*, 7206.
7. Crane, B.M., Nichols, E., Carlson, M.C., Deal, J.A., and Gross, A.L. (2023). Body Mass Index and Cognition: Associations Across Mid- to Late Life and Gender Differences. J. Gerontol. A Biol. Sci. Med. Sci. *78*, 988–996.
8. Liu, Z., Yang, H., Chen, S., Cai, J., and Huang, Z. (2019). The association between body mass index, waist circumference, waist-hip ratio and cognitive disorder in older adults. J. Public Health *41*, 305–312.
9. Liu, X., Chen, X., Hou, L., Xia, X., Hu, F., Luo, S., Zhang, G., and Dong, B. (2021). Associations of Body Mass Index, Visceral Fat Area, Waist Circumference, and Waist-to-Hip Ratio with Cognitive Function in Western China: Results from WCHAT Study. J. Nutr. Health Aging *25*, 903–908.
10. Crichton, G.E., Elias, M.F., Davey, A., Sullivan, K.J., and Robbins, M.A. (2014). Higher HDL cholesterol is associated with better cognitive function: the Maine-Syracuse study. J. Int. Neuropsychol. Soc. *20*, 961–970.
11. Svensson, T., Sawada, N., Mimura, M., Nozaki, S., Shikimoto, R., and Tsugane, S. (2019). The association between midlife serum high-density lipoprotein and mild cognitive impairment and dementia after 19 years of follow-up. Transl. Psychiatry *9*, 26.
12. Pang, K., Liu, C., Tong, J., Ouyang, W., Hu, S., and Tang, Y. (2022). Higher Total Cholesterol Concentration May Be Associated with Better Cognitive Performance among Elderly Females. Nutrients *14*, 4198.
13. Adab, P., Pallan, M., and Whincup, P.H. (2018). Is BMI the best measure of obesity? BMJ *360*, k1274.
14. Romero-Corral, A., Somers, V.K., Sierra-Johnson, J., Jensen, M.D., Thomas, R.J., Squires, R.W., Allison, T.G., Korinek, J.,

and Lopez-Jimenez, F. (2007). Diagnostic performance of body mass index to detect obesity in patients with coronary artery disease. Eur. Heart J. *28*, 2087–2093.
15. Okorodudu, D.O., Jumean, M.F., Montori, V.M., Romero-Corral, A., Somers, V.K., Erwin, P.J., and Lopez-Jimenez, F. (2010). Diagnostic performance of body mass index to identify obesity as defined by body adiposity: a systematic review and meta-analysis. Int. J. Obes. *34*, 791–799.
16. Battram, T., Yousefi, P., Crawford, G., Prince, C., Sheikhali Babaei, M., Sharp, G., Hatcher, C., Vega-Salas, M.J., Khodabakhsh, S., Whitehurst, O., et al. (2022). The EWAS Catalog: a database of epigenome-wide association studies. Wellcome Open Res. *7*, 41.
17. Pfeiffer, L., Wahl, S., Pilling, L.C., Reischl, E., Sandling, J.K., Kunze, S., Holdt, L.M., Kretschmer, A., Schramm, K., Adamski, J., et al. (2015). DNA methylation of lipid-related genes affects blood lipid levels. Circ. Cardiovasc. Genet. *8*, 334–342.
18. Kriebel, J., Herder, C., Rathmann, W., Wahl, S., Kunze, S., Molnos, S., Volkova, N., Schramm, K., Carstensen-Kirberg, M., Waldenberger, M., et al. (2016). Association between DNA Methylation in Whole Blood and Measures of Glucose Metabolism: KORA F4 Study. PLoS One *11*, e0152314.
19. Justice, A.E., Chittoor, G., Gondalia, R., Melton, P.E., Lim, E., Grove, M.L., Whitsel, E.A., Liu, C.T., Cupples, L.A., Fernandez-Rhodes, L., et al. (2020). Methylome-wide association study of central adiposity implicates genes involved in immune and endocrine systems. Epigenomics *12*, 1483–1499.
20. Sayols-Baixeras, S., Subirana, I., Fernández-Sanlés, A., Sentí, M., Lluís-Ganella, C., Marrugat, J., and Elosua, R. (2017). DNA methylation and obesity traits: An epigenome-wide association study. The REGICOR study. Epigenetics *12*, 909–916.
21. Demerath, E.W., Guan, W., Grove, M.L., Aslibekyan, S., Mendelson, M., Zhou, Y.H., Hedman, Å.K., Sandling, J.K., Li, L.A., Irvin, M.R., et al. (2015). Epigenome-wide association study (EWAS) of BMI, BMI change and waist circumference in African American adults identifies multiple replicated loci. Hum. Mol. Genet. *24*, 4464–4479.
22. Braun, K.V.E., Dhana, K., de Vries, P.S., Voortman, T., van Meurs, J.B.J., Uitterlinden, A.G., BIOS consortium, Hofman, A., Hu, F.B., Franco, O.H., and Dehghan, A. (2017). Epigenome-wide association study (EWAS) on lipids: the Rotterdam Study. Clin. Epigenetics *9*, 15.
23. Wahl, S., Drong, A., Lehne, B., Loh, M., Scott, W.R., Kunze, S., Tsai, P.C., Ried, J.S., Zhang, W., Yang, Y., et al. (2017). Epigenome-wide association study of body mass index, and the adverse outcomes of adiposity. Nature *541*, 81–86.
24. Aslibekyan, S., Demerath, E.W., Mendelson, M., Zhi, D., Guan, W., Liang, L., Sha, J., Pankow, J.S., Liu, C., Irvin, M.R., et al. (2015). Epigenome-wide study identifies novel methylation loci associated with body mass index and waist circumference. Obesity *23*, 1493–1501.
25. Geurts, Y.M., Dugué, P.A., Joo, J.E., Makalic, E., Jung, C.H., Guan, W., Nguyen, S., Grove, M.L., Wong, E.M., Hodge, A.M., et al. (2018). Novel associations between blood DNA methylation and body mass index in middle-aged and older adults. Int. J. Obes. *42*, 887–896.
26. Sharp, G.C., Alfano, R., Ghantous, A., Urquiza, J., Rifas-Shiman, S.L., Page, C.M., Jin, J., Fernández-Barrés, S., Santorelli, G., Tindula, G.; and 36 other members of the Pregnancy and Childhood Epigenetics PACE consortium (2021). Paternal body mass index and offspring DNA methylation: findings from the PACE consortium. Int. J. Epidemiol. *50*, 1297–1315.
27. Vehmeijer, F.O.L., Küpers, L.K., Sharp, G.C., Salas, L.A., Lent, S., Jima, D.D., Tindula, G., Reese, S., Qi, C., Gruzieva, O., et al. (2020). DNA methylation and body mass index from birth to adolescence: meta-analyses of epigenome-wide association studies. Genome Med. *12*, 105.
28. Liu, J., Carnero-Montoro, E., van Dongen, J., Lent, S., Nedeljkovic, I., Ligthart, S., Tsai, P.C., Martin, T.C., Mandaviya, P.R., Jansen, R., et al. (2019). An integrative cross-omics analysis of DNA methylation sites of glucose and insulin homeostasis. Nat. Commun. *10*, 2581.
29. Lim, I.Y., Lin, X., Teh, A.L., Wu, Y., Chen, L., He, M., Chan, S.Y., MacIsaac, J.L., Chan, J.K.Y., Tan, K.H., et al. (2022). Dichotomy in the Impact of Elevated Maternal Glucose Levels on Neonatal Epigenome. J. Clin. Endocrinol. Metab. *107*, e1277–e1292.
30. Antoun, E., Kitaba, N.T., Titcombe, P., Dalrymple, K.V., Garratt, E.S., Barton, S.J., Murray, R., Seed, P.T., Holbrook, J.D., Kobor, M.S., et al. (2020). Maternal dysglycaemia, changes in the infant's epigenome modified with a diet and physical activity intervention in pregnancy: Secondary analysis of a randomised control trial. PLoS Med. *17*, e1003229.
31. Ouidir, M., Zeng, X., Workalemahu, T., Shrestha, D., Grantz, K.L., Mendola, P., Zhang, C., and Tekola-Ayele, F. (2020). Early pregnancy dyslipidemia is associated with placental DNA methylation at loci relevant for cardiometabolic diseases. Epigenomics *12*, 921–934.
32. Hedman, Å.K., Mendelson, M.M., Marioni, R.E., Gustafsson, S., Joehanes, R., Irvin, M.R., Zhi, D., Sandling, J.K., Yao, C., Liu, C., et al. (2017). Epigenetic Patterns in Blood Associated With Lipid Traits Predict Incident Coronary Heart Disease Events and Are Enriched for Results From Genome-Wide Association Studies. Circ. Cardiovasc. Genet. *10*, e001487.
33. Do, W.L., Sun, D., Meeks, K., Dugué, P.-A., Demerath, E., Guan, W., Li, S., Chen, W., Milne, R., Adeyemo, A., et al. (2023). Epigenome-wide meta-analysis of BMI in nine cohorts: Examining the utility of epigenetically predicted BMI. Am. J. Hum. Genet. *110*, 273–283.
34. McCartney, D.L., Hillary, R.F., Stevenson, A.J., Ritchie, S.J., Walker, R.M., Zhang, Q., Morris, S.W., Bermingham, M.L., Campbell, A., Murray, A.D., et al. (2018). Epigenetic prediction of complex traits and death. Genome Biol. *19*, 136.
35. Stevenson, A.J., McCartney, D.L., Hillary, R.F., Campbell, A., Morris, S.W., Bermingham, M.L., Walker, R.M., Evans, K.L., Boutin, T.S., Hayward, C., et al. (2020). Characterisation of an inflammation-related epigenetic score and its association with cognitive ability. Clin. Epigenetics *12*, 113.
36. Green, C., Shen, X., Stevenson, A.J., Conole, E.L.S., Harris, M.A., Barbu, M.C., Hawkins, E.L., Adams, M.J., Hillary, R.F., Lawrie, S.M., et al. (2021). Structural brain correlates of serum and epigenetic markers of inflammation in major depressive disorder. Brain Behav. Immun. *92*, 39–48.
37. Hamilton, O.K.L., Zhang, Q., McRae, A.F., Walker, R.M., Morris, S.W., Redmond, P., Campbell, A., Murray, A.D., Porteous, D.J., Evans, K.L., et al. (2019). An epigenetic score for BMI based on DNA methylation correlates with poor physical health and major disease in the Lothian Birth Cohort. Int. J. Obes. *43*, 1795–1802.
38. Smith, B.H., Campbell, A., Linksted, P., Fitzpatrick, B., Jackson, C., Kerr, S.M., Deary, I.J., MacIntyre, D.J., Campbell, H., McGilchrist, M., et al. (2013). Cohort Profile: Generation Scotland: Scottish Family Health Study (GS:SFHS). The study, its

participants and their potential for genetic research on health and illness. Int. J. Epidemiol. *42*, 689–700.
39. Taylor, A.M., Pattie, A., and Deary, I.J. (2018). Cohort Profile Update: The Lothian Birth Cohorts of 1921 and 1936. Int. J. Epidemiol. *47*, 1042-1042r.
40. Deary, I.J., Gow, A.J., Pattie, A., and Starr, J.M. (2012). Cohort profile: the Lothian Birth Cohorts of 1921 and 1936. Int. J. Epidemiol. *41*, 1576–1584.
41. Hillary, R.F., McCartney, D.L., Smith, H.M., Bernabeu, E., Gadd, D.A., Chybowska, A.D., Cheng, Y., Murphy, L., Wrobel, N., Campbell, A., et al. (2023). Blood-based epigenome-wide analyses of 19 common disease states: A longitudinal, population-based linked cohort study of 18,413 Scottish individuals. PLoS Med. *20*, e1004247.
42. Pidsley, R., Y Wong, C.C., Volta, M., Lunnon, K., Mill, J., and Schalkwyk, L.C. (2013). A data-driven approach to preprocessing Illumina 450K methylation array data. BMC Genom. *14*, 293.
43. Zhang, Q., Marioni, R.E., Robinson, M.R., Higham, J., Sproul, D., Wray, N.R., Deary, I.J., McRae, A.F., and Visscher, P.M. (2018). Genotype effects contribute to variation in longitudinal methylome patterns in older people. Genome Med. *10*, 75.
44. Aryee, M.J., Jaffe, A.E., Corrada-Bravo, H., Ladd-Acosta, C., Feinberg, A.P., Hansen, K.D., and Irizarry, R.A. (2014). Minfi: a flexible and comprehensive Bioconductor package for the analysis of Infinium DNA methylation microarrays. Bioinformatics *30*, 1363–1369.
45. Zhang, F., Chen, W., Zhu, Z., Zhang, Q., Nabais, M.F., Qi, T., Deary, I.J., Wray, N.R., Visscher, P.M., McRae, A.F., and Yang, J. (2019). OSCA: a tool for omic-data-based complex trait analysis. Genome Biol. *20*, 107.
46. Bollepalli, S., Korhonen, T., Kaprio, J., Anders, S., and Ollikainen, M. (2019). EpiSmokEr: a robust classifier to determine smoking status from DNA methylation data. Epigenomics *11*, 1469–1486.
47. Zeilinger, S., Kühnel, B., Klopp, N., Baurecht, H., Kleinschmidt, A., Gieger, C., Weidinger, S., Lattka, E., Adamski, J., Peters, A., et al. (2013). Tobacco Smoking Leads to Extensive Genome-Wide Changes in DNA Methylation. PLoS One *8*, e63812.
48. Houseman, E.A., Accomando, W.P., Koestler, D.C., Christensen, B.C., Marsit, C.J., Nelson, H.H., Wiencke, J.K., and Kelsey, K.T. (2012). DNA methylation arrays as surrogate measures of cell mixture distribution. BMC Bioinf. *13*, 86.
49. van Iterson, M., van Zwet, E.W., BIOS Consortium, and Heijmans, B.T. (2017). Controlling bias and inflation in epigenome- and transcriptome-wide association studies using the empirical null distribution. Genome Biol. *18*, 19.
50. Saffari, A., Silver, M.J., Zavattari, P., Moi, L., Columbano, A., Meaburn, E.L., and Dudbridge, F. (2018). Estimation of a significance threshold for epigenome-wide association studies. Genet. Epidemiol. *42*, 20–33.
51. Pedregosa, F., Varoquaux, G., Gramfort, A., Michel, V., Thirion, B., Grisel, O., Blondel, M., Prettenhofer, P., Weiss, R., Dubourg, V., et al. (2011). Scikit-learn: Machine Learning in Python. J. Mach. Learn. Res. *12*, 2825–2830.
52. Trejo Banos, D., McCartney, D.L., Patxot, M., Anchieri, L., Battram, T., Christiansen, C., Costeira, R., Walker, R.M., Morris, S.W., Campbell, A., et al. (2020). Bayesian reassessment of the epigenetic architecture of complex traits. Nat. Commun. *11*, 2865.
53. Zeng, Y., and Breheny, P. (2017). The biglasso Package: A Memory- and Computation-Efficient Solver for Lasso Model Fitting with Big Data in R. Preprint at arXiv. https://arxiv.org/abs/1701.05936.
54. Rosseel, Y. (2012). lavaan: An R Package for Structural Equation Modeling. J. Stat. Softw. *48*, 1–36.
55. Mendelson, M.M., Marioni, R.E., Joehanes, R., Liu, C., Hedman, Å.K., Aslibekyan, S., Demerath, E.W., Guan, W., Zhi, D., Yao, C., et al. (2017). Association of Body Mass Index with DNA Methylation and Gene Expression in Blood Cells and Relations to Cardiometabolic Disease: A Mendelian Randomization Approach. PLoS Med. *14*, e1002215.
56. Shah, S., Bonder, M.J., Marioni, R.E., Zhu, Z., McRae, A.F., Zhernakova, A., Harris, S.E., Liewald, D., Henders, A.K., Mendelson, M.M., et al. (2015). Improving Phenotypic Prediction by Combining Genetic and Epigenetic Associations. Am. J. Hum. Genet. *97*, 75–85.
57. Sun, D., Zhang, T., Su, S., Hao, G., Chen, T., Li, Q.Z., Bazzano, L., He, J., Wang, X., Li, S., and Chen, W. (2019). Body Mass Index Drives Changes in DNA Methylation: A Longitudinal Study. Circ. Res. *125*, 824–833.
58. Matsuo, M. (2022). ABCA1 and ABCG1 as potential therapeutic targets for the prevention of atherosclerosis. J. Pharmacol. Sci. *148*, 197–203.
59. Kobayashi, A., Takanezawa, Y., Hirata, T., Shimizu, Y., Misasa, K., Kioka, N., Arai, H., Ueda, K., and Matsuo, M. (2006). Efflux of sphingomyelin, cholesterol, and phosphatidylcholine by ABCG1. J. Lipid Res. *47*, 1791–1802.
60. Schlaepfer, I.R., and Joshi, M. (2020). CPT1A-mediated Fat Oxidation, Mechanisms, and Therapeutic Potential. Endocrinology *161*, bqz046.
61. Sugden, M.C., and Holness, M.J. (2006). Mechanisms underlying regulation of the expression and activities of the mammalian pyruvate dehydrogenase kinases. Arch. Physiol. Biochem. *112*, 139–149.
62. Pettersen, I.K.N., Tusubira, D., Ashrafi, H., Dyrstad, S.E., Hansen, L., Liu, X.-Z., Nilsson, L.I.H., Løvsletten, N.G., Berge, K., Wergedahl, H., et al. (2019). Upregulated PDK4 expression is a sensitive marker of increased fatty acid oxidation. Mitochondrion *49*, 97–110.
63. Dekkers, K.F., van Iterson, M., Slieker, R.C., Moed, M.H., Bonder, M.J., van Galen, M., Mei, H., Zhernakova, D.V., van den Berg, L.H., Deelen, J., et al. (2016). Blood lipids influence DNA methylation in circulating cells. Genome Biol. *17*, 138.
64. Wulan, S.N., Westerterp, K.R., and Plasqui, G. (2010). Ethnic differences in body composition and the associated metabolic profile: A comparative study between Asians and Caucasians. Maturitas *65*, 315–319.
65. Rush, E.C., Freitas, I., and Plank, L.D. (2009). Body size, body composition and fat distribution: comparative analysis of European, Maori, Pacific Island and Asian Indian adults. Br. J. Nutr. *102*, 632–641.
66. Merzbacher, C., Ryan, B., Goldsborough, T., Hillary, R.F., Campbell, A., Murphy, L., McIntosh, A.M., Liewald, D., Harris, S.E., McRae, A.F., et al. (2023). Integration of datasets for individual prediction of DNA methylation-based biomarkers. Genome Biol. *24*, 278.
67. Cheng, Y., Gieger, C., Campbell, A., McIntosh, A.M., Waldenberger, M., McCartney, D.L., Marioni, R.E., and Vallejos, C.A. (2024). Feature pre-selection for the development of epigenetic biomarkers. Preprint at medRxiv. https://doi.org/10.1101/2024.02.14.24302694.

The American Journal of Human Genetics, Volume *112*

## Supplemental information

# DNA methylation-based predictors of metabolic traits in Scottish and Singaporean cohorts

**Hannah M. Smith, Hong Kiat Ng, Joanna E. Moodie, Danni A. Gadd, Daniel L. McCartney, Elena Bernabeu, Archie Campbell, Paul Redmond, Adele Taylor, Danielle Page, Janie Corley, Sarah E. Harris, Darwin Tay, Ian J. Deary, Kathryn L. Evans, Matthew R. Robinson, John C. Chambers, Marie Loh, Simon R. Cox, Riccardo E. Marioni, and Robert F. Hillary**

A)

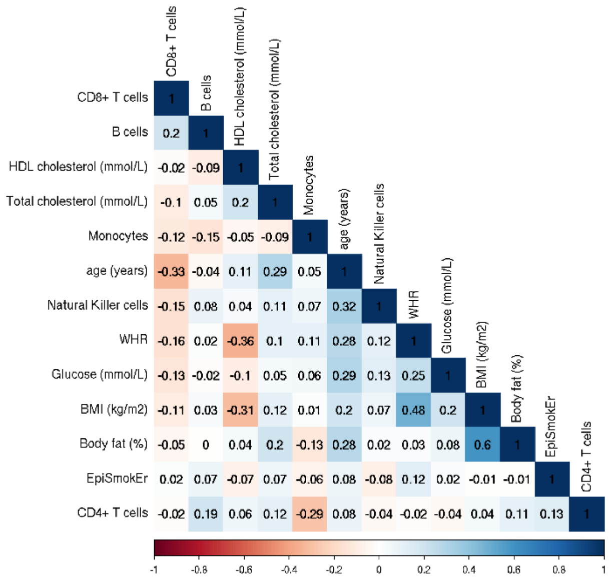


B)

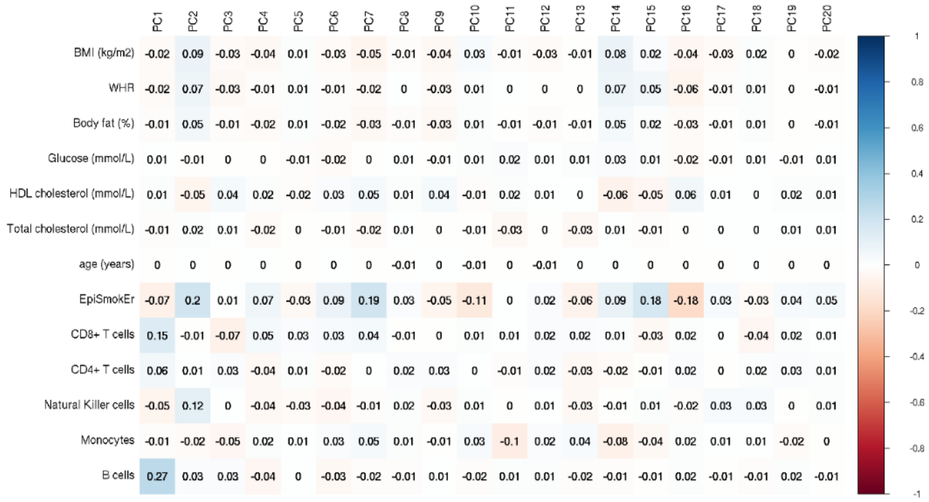


**Figure S1: Metabolic trait, covariate and 20 DNAm PCs correlations in Generation Scotland.** Figure S1A shows a heatmap of Pearson correlations between metabolic traits (BMI in kg/m$^2$; HDL cholesterol, total cholesterol and glucose in mmol/L; body fat in percentage; WHR) and covariates in Generation Scotland. Figure S1B shows a heatmap of Pearson correlations between metabolic traits/covariates and the first 20 DNAm PCs. BMI = body mass index; WHR = waist-hip ratio; HDL cholesterol = high-density lipoprotein cholesterol; EpiSmokEr = epigenetic smoking score; DNAm = DNA methylation; PCs = principal components.

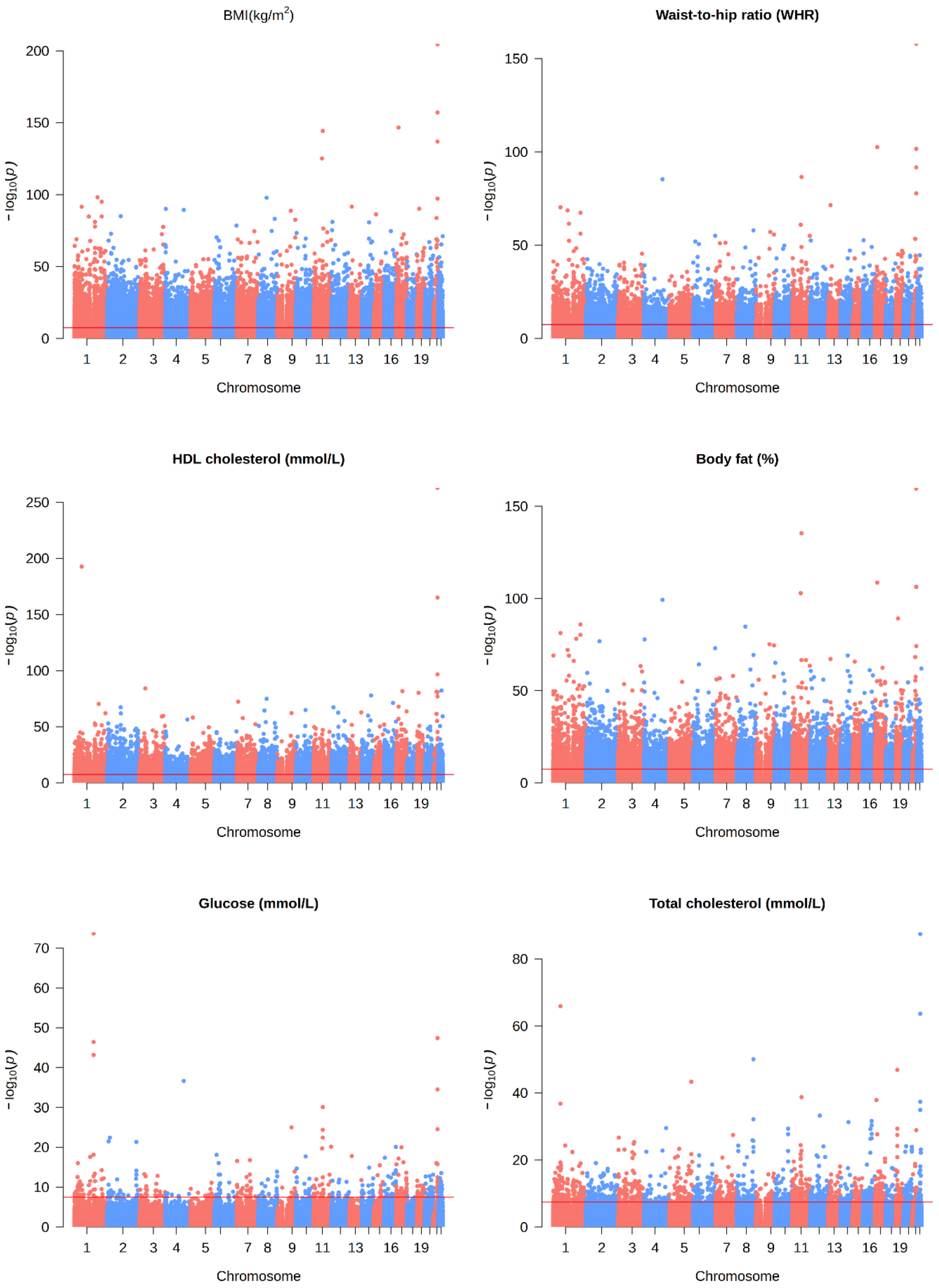


**Figure S2: Manhattan plots of the results from the non-PC-adjusted marginal linear regression epigenome-wide association studies of six metabolic traits in Generation Scotland.** The Manhattan plots for each of the six metabolic traits show each CpG as a data point. Outcomes in each EWAS are the residuals from metabolic traits regressed on age, age$^2$, sex and family structure. Original outcome units are indicated in the plot titles. The x-axis shows the chromosome position, and the y-axis shows the association significance ($-\log_{10}(P)$) for each CpG site. The horizontal red line indicates the significance threshold ($P < 3.6 \times 10^{-8}$). BMI =

body mass index; WHR = waist-hip ratio; HDL cholesterol = high-density lipoprotein cholesterol; PC = principal component.

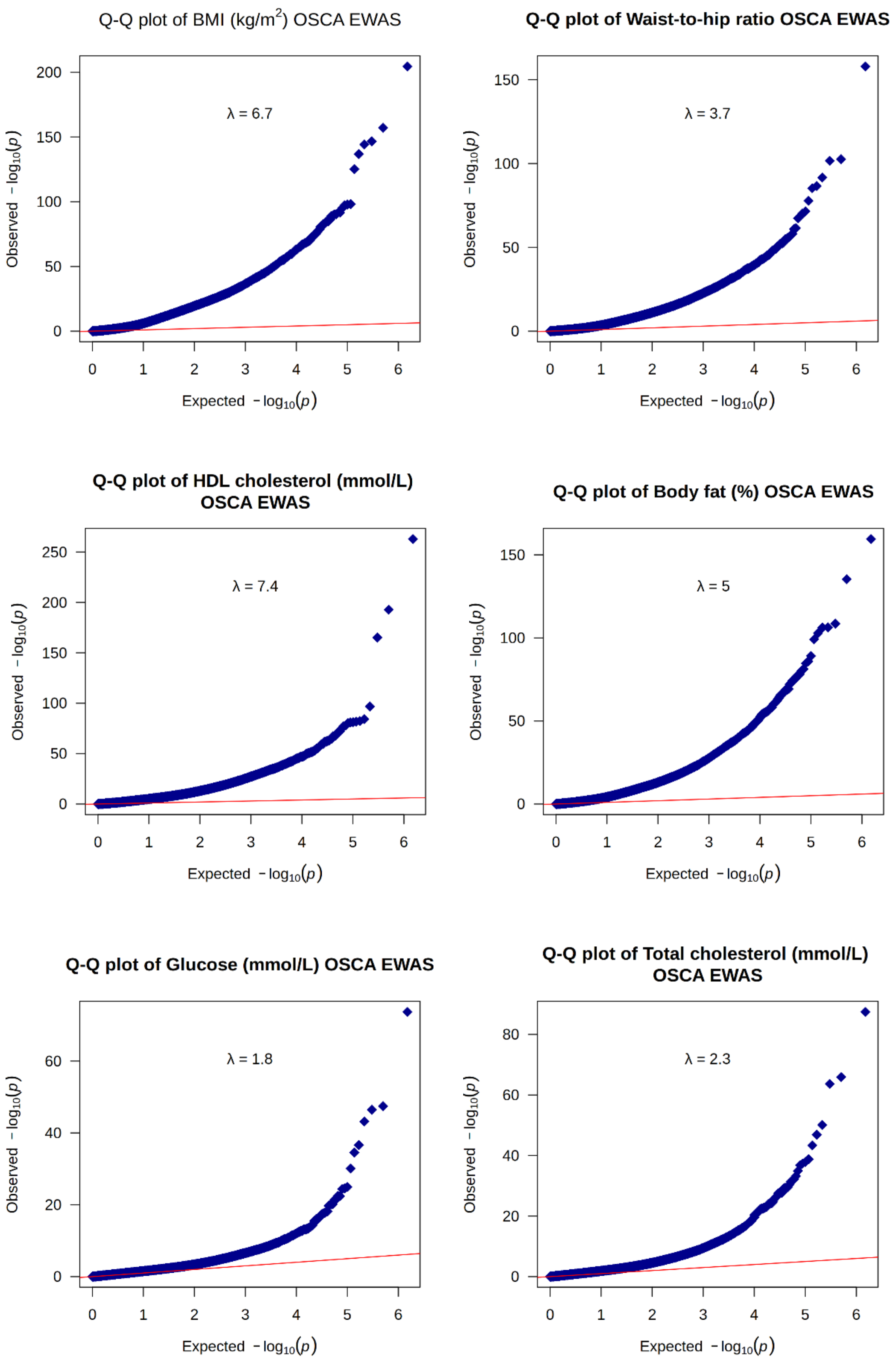

**Figure S3: Quantile-Quantile plots of the results from the non-PC-adjusted marginal linear regression epigenome-wide association studies of six metabolic traits in Generation Scotland.** The plots show expected $-\log_{10}(P)$ by the observed $-\log_{10}(P)$ for each metabolic trait. Outcomes in each EWAS are the residuals from metabolic traits regressed on age, $age^2$, sex and family structure. Original outcome units are indicated in the plot titles. The red line shows a trend line of where the observed and expected values are the same. The inflation factor, lambda ($\lambda$), is indicated on each plot. BMI = body mass index; WHR = waist-hip ratio; HDL cholesterol = high-density lipoprotein cholesterol; PC = principal component.

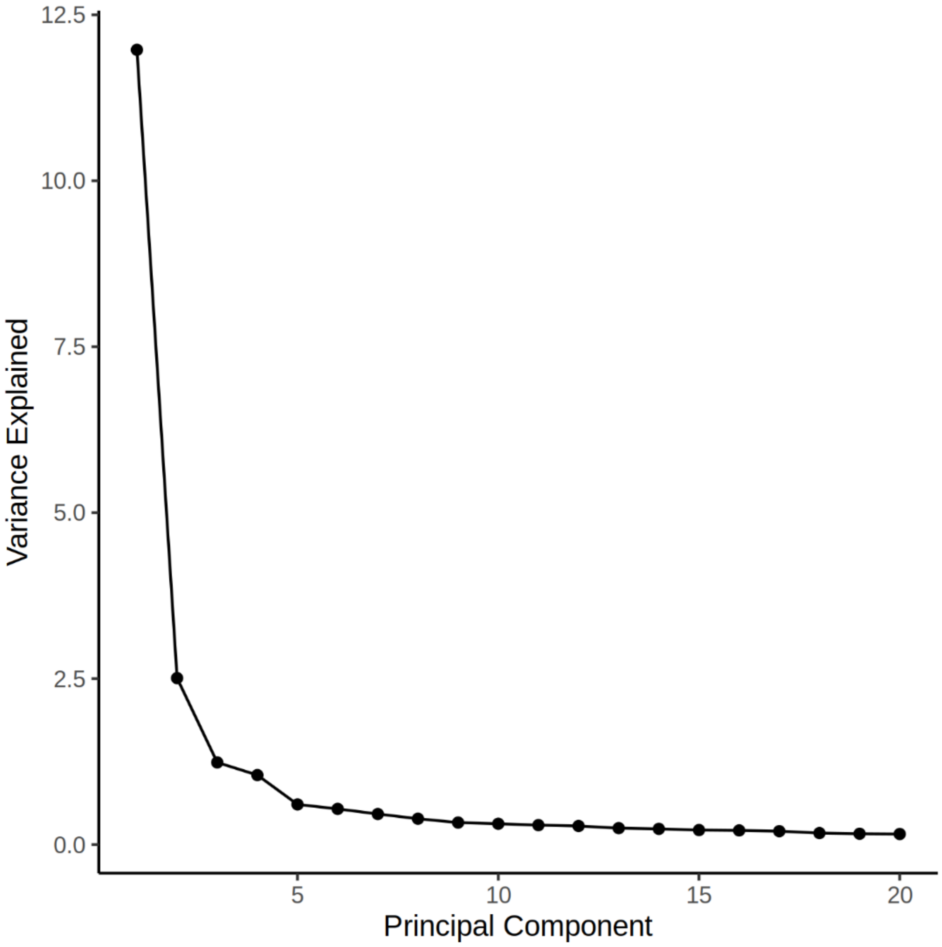


**Figure S4: Variance explained in DNA methylation by the first 20 principal components.** The plot shows the variance explained in the DNA methylation data by each of the first 20 principal components in Generation Scotland.

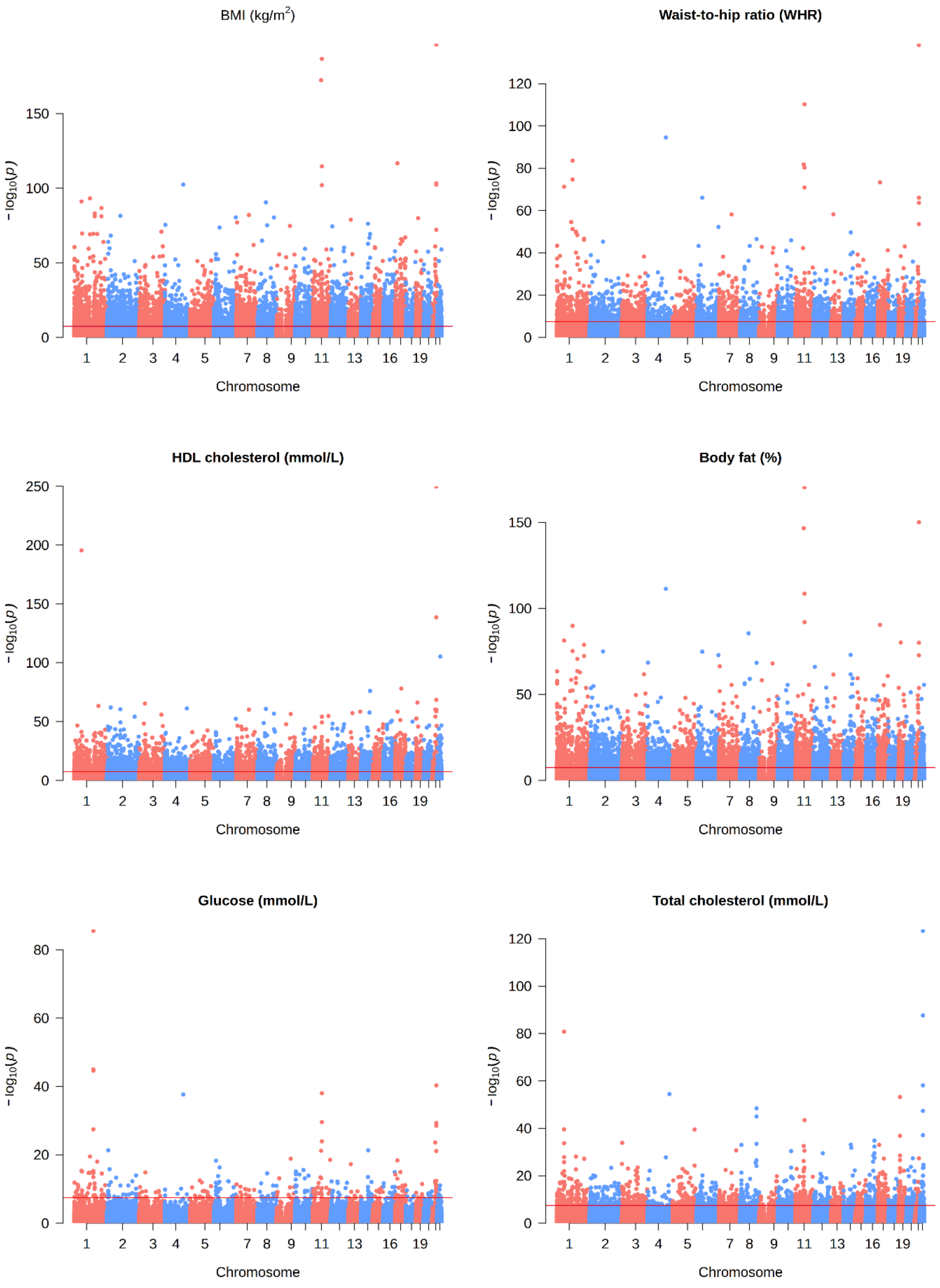


**Figure S5: Manhattan plots of the results from the DNAm-PC-adjusted marginal linear regression epigenome-wide association studies of six metabolic traits in Generation Scotland.** The Manhattan plots for each of the six metabolic traits show each CpG as a data point. Outcomes in each EWAS are the residuals from metabolic traits regressed on age, age$^2$, sex and family structure. Original outcome units are indicated in the plot titles. The x-axis shows the chromosome position, and the y-axis shows the association significance (-$\log_{10}$(P)) for each CpG site. The horizontal red line indicates the significance threshold ($P < 3.6 \times 10^{-8}$). BMI =

body mass index; WHR = waist-hip ratio; HDL cholesterol = high-density lipoprotein cholesterol; DNAm = DNA methylation; PC = principal component.

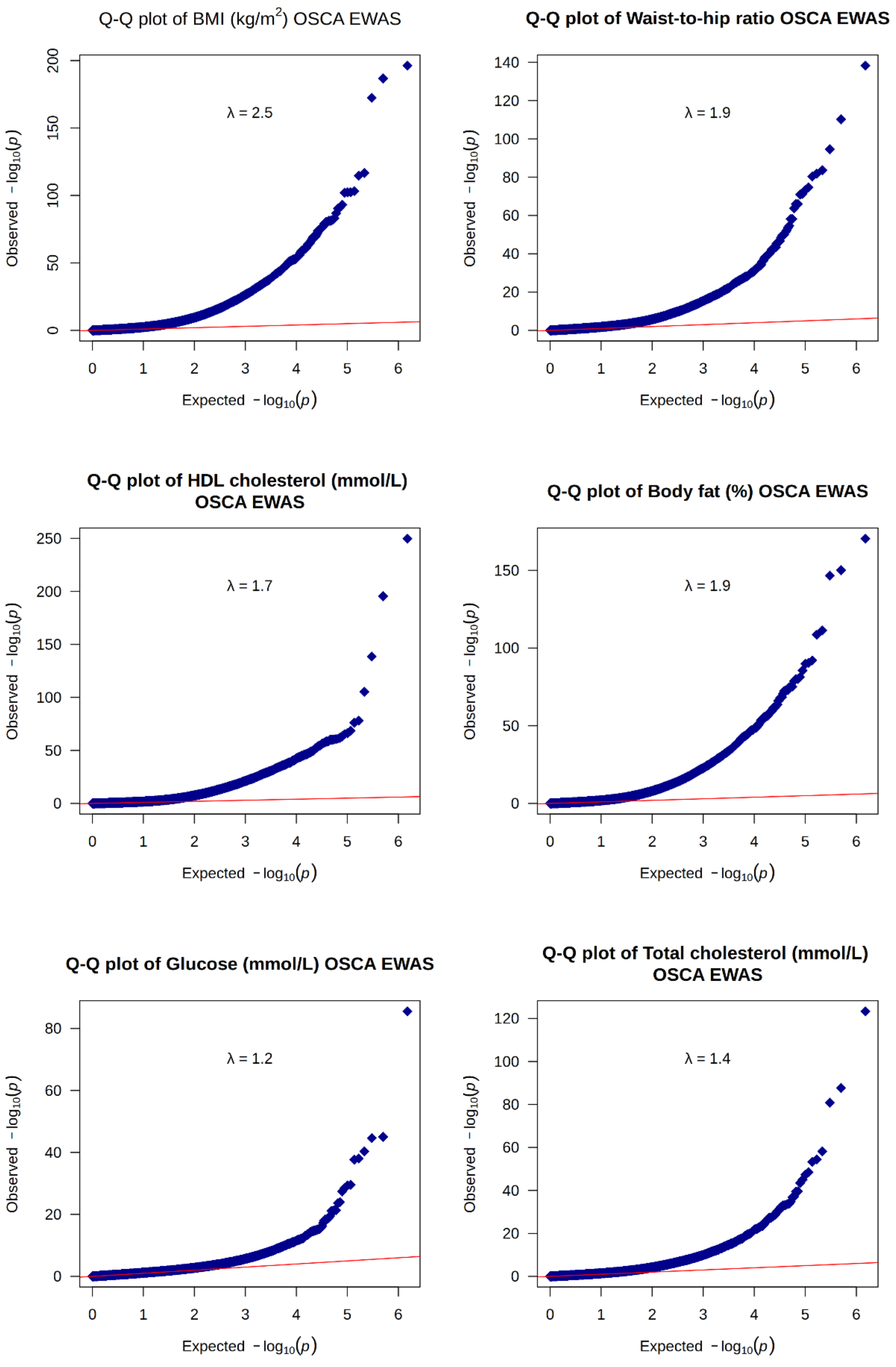


**Figure S6: Quantile-Quantile plots of the results from the DNAm-PC-adjusted marginal linear regression epigenome-wide association studies of six metabolic traits in Generation Scotland.** The plots show

expected –log$_{10}$(P) by the observed –log$_{10}$(P) for each metabolic trait. Outcomes in each EWAS are the residuals from metabolic traits regressed on age, age$^2$, sex and family structure. Original outcome units are indicated in the plot titles The red line shows a trend line of where the observed and expected values are the same. The inflation factor, lambda (λ), is indicated on each plot. BMI = body mass index; WHR = waist-hip ratio; HDL cholesterol = high-density lipoprotein cholesterol; DNAm = DNA methylation; PC = principal component.

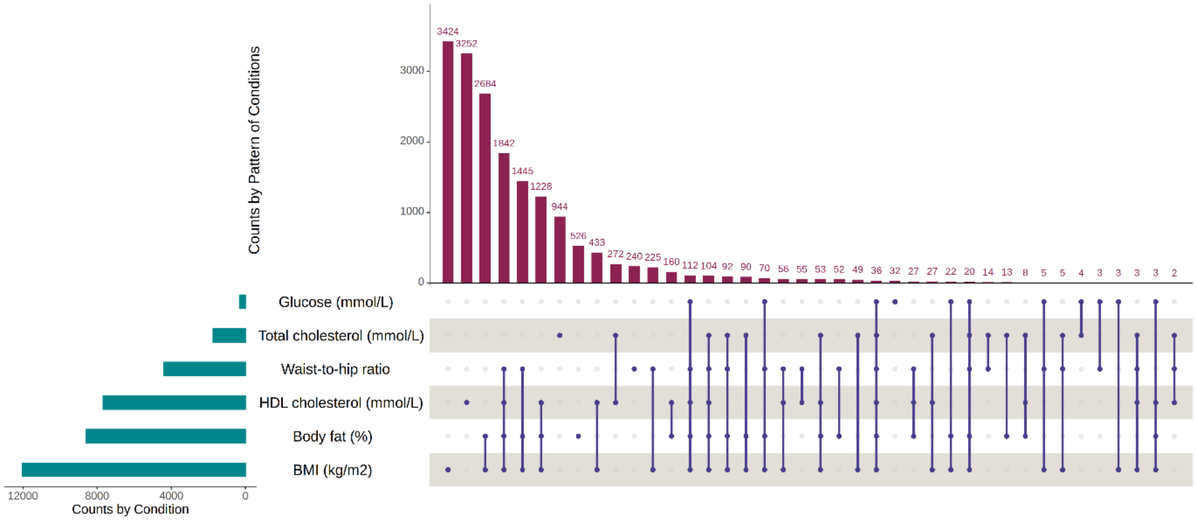


**Figure S7: An upset plot of significant CpGs for the six metabolic traits in Generation Scotland.** The figure shows the number of unique and overlapping CpGs for the six metabolic traits from the DNAm-PC-adjusted marginal linear regression models. Outcomes in each EWAS are the residuals from metabolic traits regressed on age, age$^2$, sex and family structure. Original outcome units are indicated in the plot. BMI = body mass index; WHR = waist-hip ratio; HDL cholesterol = high-density lipoprotein cholesterol; DNAm = DNA methylation; PC = principal component.

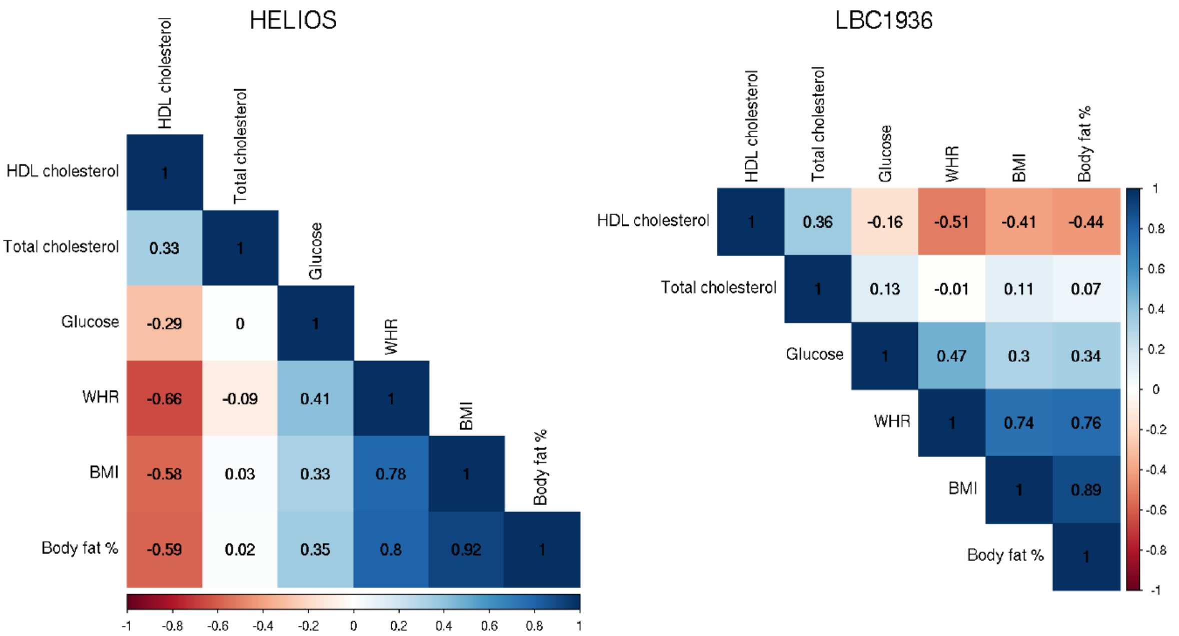


**Figure S8: Metabolic EpiScore correlations for the Health for Life in Singapore (HELIOS) study and the Lothian Birth Cohort 1936 (LBC1936).** The heatmaps show the Pearson correlation between each metabolic EpiScore in HELIOS and LBC1936. BMI = body mass index; WHR = waist-hip ratio; HDL cholesterol = high-density lipoprotein cholesterol.

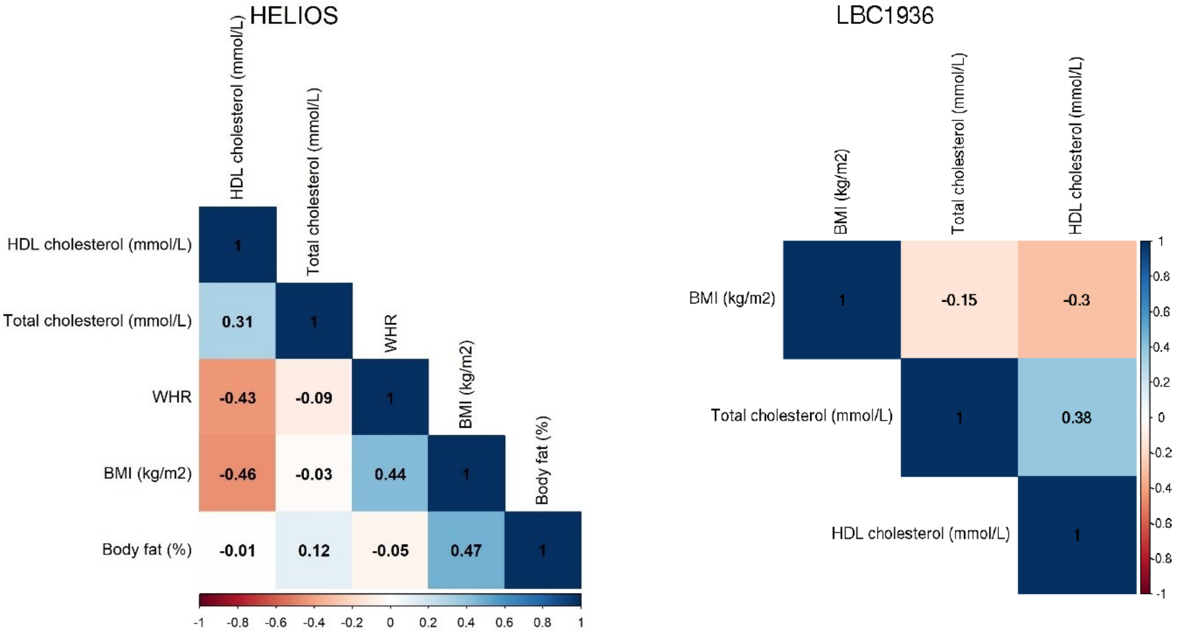

**Figure S9: Metabolic trait correlations in the Health for Life in Singapore (HELIOS) study and the Lothian Birth Cohort 1936 (LBC1936).** The heatmaps show the Pearson correlation between measured metabolic traits in HELIOS and LBC1936. BMI = body mass index; WHR = waist-hip ratio; HDL cholesterol = high-density lipoprotein cholesterol.

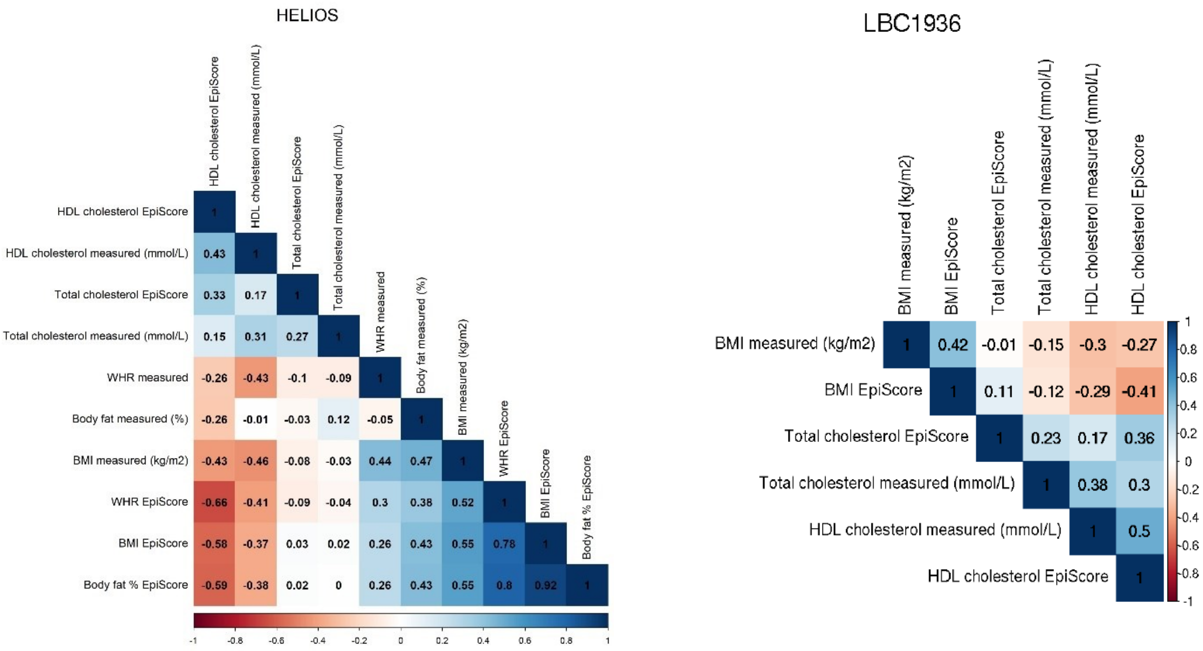


**Figure S10: EpiScore-metabolic trait correlations in the Health for Life in Singapore (HELIOS) and the Lothian Birth Cohort (LBC1936).** The heatmaps show the Pearson correlations between metabolic EpiScores and measured metabolic traits in HELIOS and LBC1936. BMI = body mass index; WHR = waist-hip ratio; HDL cholesterol = high-density lipoprotein cholesterol.

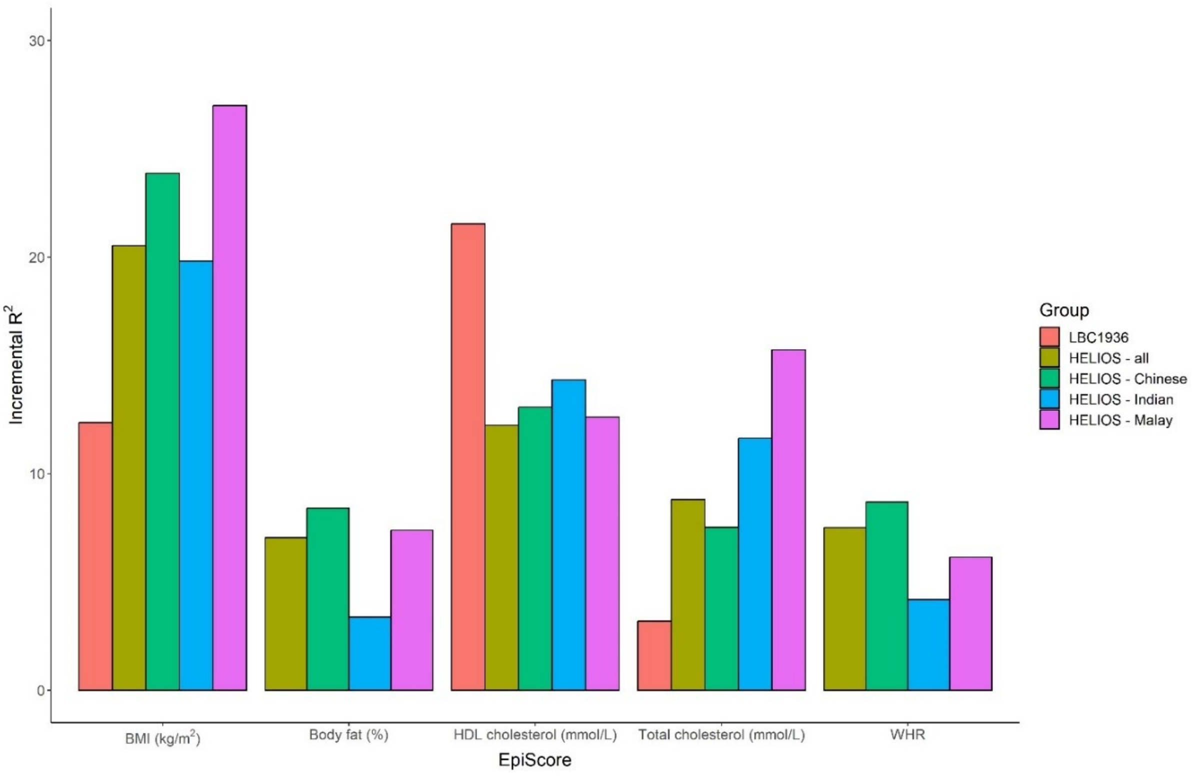


**Figure S11: The variance explained in measured metabolic traits by Bayesian EpiScores in the Health for Life in Singapore (HELIOS) study and the Lothian Birth Cohort 1936 (LBC1936).** The figure shows the incremental $R^2$ for each metabolic trait (BMI in kg/m$^2$; HDL cholesterol and total cholesterol in mmol/L; body fat in percentage; WHR) accounted for by their corresponding Bayesian metabolic EpiScores over and above age and sex-adjusted linear regression models in LBC1936 and HELIOS. The incremental $R^2$ was calculated for each subset and in the whole cohort for the HELIOS study. Full cohort models in HELIOS were additionally adjusted for subgroup (Chinese, Malay and Indian). BMI = body mass index; WHR = waist-hip ratio; HDL cholesterol = high-density lipoprotein cholesterol.

.

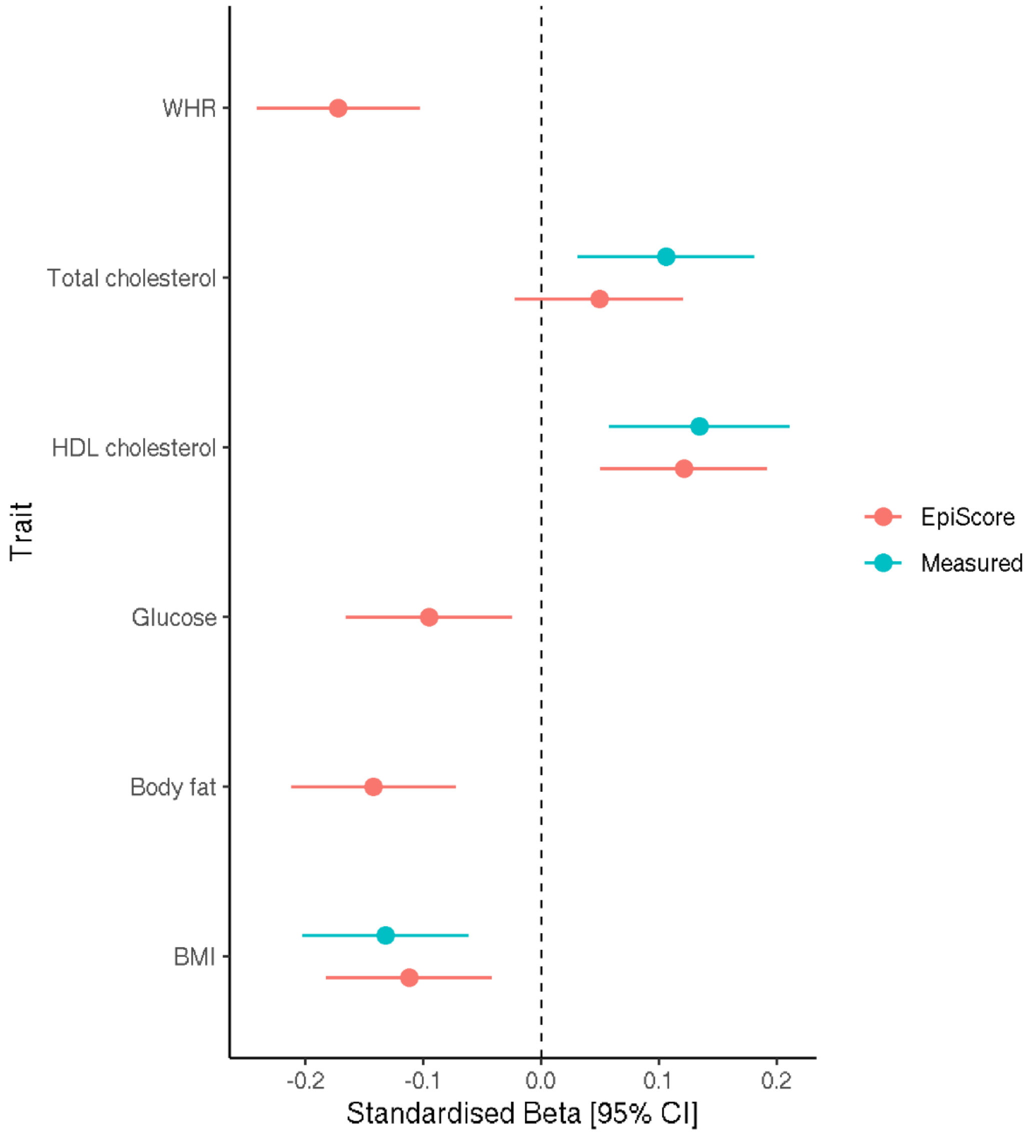


**Figure S12: EpiScore and measured metabolic trait associations with general cognitive function level in the Lothian Birth Cohort (LBC1936).** The figure shows associations between measured metabolic traits (BMI in kg/m$^2$; HDL cholesterol and total cholesterol in mmol/L) or EpiScores with general cognitive function level in models with basic adjustments (age and sex). Standardised betas are shown and error bars represent 95% confidence intervals. BMI = body mass index; WHR = waist-hip ratio; HDL cholesterol = high-density lipoprotein cholesterol.

## Supplemental Methods

### Metabolic measures in Generation Scotland (GS), Lothian Birth Cohort 1936 (LBC1936) and the Health for Life in Singapore (HELIOS) study

This study investigated six metabolic measures including body mass index (BMI), waist-hip ratio (WHR), body fat percentage, high-density lipoprotein (HDL) cholesterol, total cholesterol and glucose. The Generation Scotland (GS) study had all six traits available for analysis. The HELIOS study had all traits available for analysis except glucose. The LBC1936 had only BMI, HDL cholesterol and total cholesterol available for analysis. Outlier removal strategies were chosen on a cohort-by-cohort basis in line with previous approaches. In GS, BMI, body fat percentage and WHR outliers were removed by visual inspection after bivariate plots of all pairwise combinations ($n_{removed}$ = 426). Outliers > 4 standard deviations from the mean were removed for glucose, HDL cholesterol and total cholesterol ($n_{removed}$ = 173, 26, 15, respectively). In LBC1936, measured metabolic trait data were visually inspected and no outliers were removed. In HELIOS, data points were considered outliers if they were beyond 3.5 standard deviations from the mean. BMI (weight in kg /height in $m^2$), WHR (waist/hip circumference) and body fat percentage were measured in the clinic. In HELIOS, whole-body DEXA scans were used to quantify body fat [1]. In GS, body fat percentage is quantified with bioimpedance. HDL cholesterol, total cholesterol and glucose from blood samples were measured in mmol/L.

**Bayesian EWAS**

BayesR+ is a software implemented in C++ for performing Bayesian penalised regression on complex continuous traits [2]. A prior distribution is assumed as a mixture of Gaussian distributions, which correspond to groups of probes with different effect sizes. A discrete spike at zero is included, which removes probes that have a negligible effect on the trait. Informed by data from a previous analysis of BMI, prior mixture variances of probes were set to 0.0001, 0.001, 0.01 [2]. Pre-

corrected phenotype and DNA methylation data were scaled to mean zero and unit variance. Gibbs sampling was used to sample over the posterior distribution and consisted of 10,000 samples with 5,000 as burn-in. A thinning of 5 samples was applied to reduce autocorrelation. Four chains were used and the final 250 samples per chain (after thinning) were combined to form the final set of 1,000 iterations from which variance and effect size estimates were obtained. Probes with a posterior inclusion probability (PIP) ≥ 95% were deemed to be significant.

**Cognitive measures in the Lothian Birth Cohort 1936 (LBC1936)**

Cognitive measures in the LBC1936 have been described previously [3-6]. Cognitive testing was repeated for 5 waves at ages 70, 73, 76, 79, and 82. Thirteen cognitive measures for all five waves were available. Several cognitive domains were measured including visuospatial ability (tests: Block Design, Matrix Reasoning (WAIS-III$^{UK}$) and Spatial span (WMS-III$^{UK}$)), memory (tests: Verbal Paired Associates, Logical Memory – a combination of immediate and delayed memory (WMS-III$^{UK}$) and Digit-span backwards (WAIS-III$^{UK}$)), and verbal ability (tests: National Adult Reading Test, Wechsler Adult Reading Test and Verbal Fluency Test (using letters V, F and L)). Processing speed was evaluated using the Digit-symbol, Symbol Search (WAIS-III$^{UK}$), Choice Reaction Time and Inspection Data availability and descriptive statistics for each measure can be found in **Table S3**.

**General cognitive function level and change in LBC1936**

Latent measures of general cognitive function and change were generated using confirmatory factor analysis in a structural equation modelling (SEM) framework using the *Lavaan* (version 0.6-12) R package [7]. Intercepts and slopes of each cognitive test were used to indicate a latent intercept and slope (level and change) of general

cognitive function (**Table S4**). Levels and changes in cognitive functioning were modelled with a latent growth curve model (LGCM) using a Factor of Curves specification [8]. A first-order hierarchical structure was specified, and residual covariance between tests in the same cognitive domain was included, in line with a previously established correlational structure of cognitive domains (speed, memory, verbal ability and visuospatial [9]). Residual covariance between intercept and slope for individual tests was also modelled. Within-wave residual covariance between the National Adult Reading Test and the Wechsler Adult Reading Test were modelled as these tests were highly correlated. The marker method was used to scale according to the first variable to aid model convergence. Negative latent residual variances were fixed to zero. Full information maximum likelihood was used to include all data available. Confirmatory factor index (CFI), Tucker-Lewis index (TLI), root mean squared error approximation (RMSEA) and the standardised root mean squared residual (SRMR) fit measures are reported (**Table S5**). Linear regression models were run in Lavaan to test associations between general cognitive function level and change, and the metabolic traits/EpiScores with basic- and full-adjustments as follows:

*Basic model: Latent G factor (intercept or slope) ~ measured trait/EpiScore + Age at baseline + Sex*

*Full model: Latent G factor (intercept or slope) ~ measured trait/EpiScore + Age at baseline + Sex + Scottish Index of Multiple Deprivation (SIMD) + Epigenetic smoking score (EpiSmokEr) + Alcohol units per week*

Descriptive statistics for each covariate in LBC1936 can be found in **Table S6**.

**Acknowledgements**

This research was funded in whole, or in part, by the Wellcome Trust (218493/Z/19/Z, 104036/Z/14/Z, 108890/Z/15/Z, and 221890/Z/20/Z). For the purpose of open access, the author has applied a CC BY public copyright license to any Author Accepted Manuscript version arising from this submission. GS received core support from the Chief Scientist Office of the Scottish Government Health Directorates (CZD/16/6) and the Scottish Funding Council (HR03006). DNA methylation profiling of the GS samples was carried out by the Genetics Core Laboratory at the Edinburgh Clinical Research Facility, Edinburgh, Scotland, and was funded by the Medical Research Council UK and Wellcome (Wellcome Trust Strategic Award STratifying Resilience and Depression Longitudinally (STRADL; Reference 104036/Z/14/Z). DNA methylation data for Generation Scotland was also funded by a 2018 NARSAD Young Investigator Grant from the Brain & Behavior Research Foundation (Ref: 27404; awardee: Dr David M Howard) and by a John, Margaret, Alfred and Stewart Sim Fellowship from the Royal College of Physicians of Edinburgh (Awardee: Dr Heather C Whalley). This work was supported by the European Union Horizon 2020 (PHC.03.15, project No 666881), SVDs@Target, the Fondation Leducq Transatlantic Network of Excellence for the Study of Perivascular Spaces in Small Vessel Disease [ref no. 16 CVD 05]. We thank the LBC1936 participants and team members who contributed to these studies. The LBC1936 is supported by the Biotechnology and Biological Sciences Research Council, and the Economic and Social Research Council [BB/W008793/1] (which supports S.E.H., J.C. and A.T.), Age UK (Disconnected Mind project), the Milton Damerel Trust, the Medical Research Council (G0701120, G1001245, MR/M013111/1, MR/R024065/1) and the University of Edinburgh. Methylation typing of LBC1936 was supported by the Centre for Cognitive Ageing and Cognitive Epidemiology (Pilot Fund award), Age UK, The Wellcome Trust Institutional Strategic

Support Fund, The University of Edinburgh, and The University of Queensland. H.M.S and D.A.G are supported by funding from the Wellcome Trust 4 year PhD in Translational Neuroscience: training the next generation of basic neuroscientists to embrace clinical research [218493/Z/19/Z,108890/Z/15/Z]. S.R.C. was supported by a National Institutes of Health (NIH) research grant R01AG054628 and is supported by a Sir Henry Dale Fellowship jointly funded by the Wellcome Trust and the Royal Society (Grant Number 221890/Z/20/Z). D.L.Mc.C. and R.E.M. are supported by Alzheimers Research UK major project grant ARUK/PG2017B/10. E.B and R.E.M. are supported by Alzheimer's Society major project grant AS-PG-19b-010. R.F.H is supported by an MRC IEU Fellowship. The HELIOS study is supported by Singapore Ministry of Health's (MOH) National Medical Research Council (NMRC) under its OF-LCG funding scheme (MOH-000271-00), Singapore Translational Research (StaR) funding scheme (NMRC/StaR/0028/2017), the National Research Foundation, Singapore through the Singapore MOH NMRC and the Precision Health Research, Singapore (PRECISE) under the National Precision Medicine programme (NMRC/PRECISE/2020) and intramural funding from Nanyang Technological University, Lee Kong Chian School of Medicine and the National Healthcare Group.

1. Mina, T., Yew, Y.W., Ng, H.K., Sadhu, N., Wansaicheong, G., Dalan, R., Low, D.Y.W., Lam, B.C.C., Riboli, E., Lee, E.S. *et al.* (2023). Adiposity impacts cognitive function in Asian populations: an epidemiological and Mendelian Randomization study. The Lancet Regional Health – Western Pacific 33.
2. Trejo Banos, D., McCartney, D.L., Patxot, M., Anchieri, L., Battram, T., Christiansen, C., Costeira, R., Walker, R.M., Morris, S.W., Campbell, A. *et al.* (2020). Bayesian reassessment of the epigenetic architecture of complex traits. Nature Communications 11, 2865.
3. Deary, I.J., Gow, A.J., Pattie, A. & Starr, J.M. (2012). Cohort profile: the Lothian Birth Cohorts of 1921 and 1936. Int J Epidemiol 41, 1576-84.
4. Taylor, A.M., Pattie, A. & Deary, I.J. (2018). Cohort Profile Update: The Lothian Birth Cohorts of 1921 and 1936. International Journal of Epidemiology 47, 1042-1042r.
5. Deary, I.J., Gow, A.J., Taylor, M.D., Corley, J., Brett, C., Wilson, V., Campbell, H., Whalley, L.J., Visscher, P.M., Porteous, D.J. *et al.* (2007). The Lothian Birth Cohort 1936: a study to

examine influences on cognitive ageing from age 11 to age 70 and beyond. BMC Geriatrics 7, 28.

6. Deary, I.J., Whiteman, M.C., Starr, J.M., Whalley, L.J. & Fox, H.C. (2004). The impact of childhood intelligence on later life: following up the Scottish mental surveys of 1932 and 1947. J Pers Soc Psychol 86, 130-47.
7. Rosseel, Y. (2012). lavaan: An R Package for Structural Equation Modeling. Journal of Statistical Software 48, 1 - 36.
8. McArdle, J.J. Dynamic but Structural Equation Modeling of Repeated Measures Data. in *Handbook of Multivariate Experimental Psychology* (eds. Nesselroade, J.R. & Cattell, R.B.) 561-614 (Springer US, Boston, MA, 1988).
9. Tucker-Drob, E.M., Briley, D.A., Starr, J.M. & Deary, I.J. (2014). Structure and correlates of cognitive aging in a narrow age cohort. Psychology and Aging 29, 236-249.
